# PML-RARα interaction with TRIB3 impedes PPARγ/RXR function and triggers dyslipidemia in acute promyelocytic leukemia

**DOI:** 10.7150/thno.45924

**Published:** 2020-08-15

**Authors:** Ke Li, Feng Wang, Zhao-Na Yang, Bing Cui, Ping-Ping Li, Zhen-Yu Li, Zhuo-Wei Hu, Hong-Hu Zhu

**Affiliations:** 1 National Clinical Research Center for Metabolic Disease, Department of Metabolism and Endocrinology, the Second Xiangya Hospital, Central South University, Changsha, Hunan, 410011, China.; 2 Department of Hematology & Institute of Hematology, Zhejiang Province Key Laboratory of Hematology Oncology Diagnosis and Treatment, The First Affiliated Hospital, Zhejiang University, Hangzhou, Zhejiang, 310058, China.; 3 Immunology and Cancer Pharmacology Group, State Key Laboratory of Bioactive Substance and Function of Natural Medicines, Institute of Materia Medica, Chinese Academy of Medical Sciences & Peking Union Medical College, Beijing, 100050, China.; 4 NHC Key Laboratory of Biotechnology of Antibiotics, Institute of Medicinal Biotechnology, Chinese Academy of Medical Sciences & Peking Union Medical College, Beijing, 100050, China.; 5 Department of Hematology, Affiliated Hospital of Xuzhou Medical University, Xuzhou, Jiangsu, 230031 China.

**Keywords:** AML, Cancer, leukemia, lipid metabolism, tribbles

## Abstract

Although dyslipidemia commonly occurs in patients with acute promyelocytic leukemia (APL) in response to anti-APL therapy, the underlying mechanism and the lipid statuses of patients with newly diagnosed APL remain to be addressed.

**Methods**: We conducted a retrospective study to investigate the lipid profiles of APL patients. PML-RARα transgenic mice and APL cells-transplanted mice were used to assess the effects of APL cells on the blood/liver lipid levels. Subsequently, gene set enrichment analysis, western blot and dual luciferase reporter assay were performed to examine the role and mechanism of PML-RARα and TRIB3 in lipid metabolism regulation in APL patients at pretreatment and after induction therapy.

**Results**: APL patients exhibited a higher prevalence of dyslipidemia before anti-APL therapy based on a retrospective study. Furthermore, APL cells caused secretion of triglycerides, cholesterol, and PCSK9 from hepatocytes and degradation of low-density lipoprotein receptors in hepatocytes, which elevated the lipid levels in APL cell-transplanted mice and *Pml-Rarα* transgenic mice. Mechanistically, pseudokinase TRIB3 interacted with PML-RARα to inhibit PPARγ activity by interfering with the interaction of PPARγ and RXR and promoting PPARγ degradation. Thus, reduced PPARγ activity in APL cells decreased leptin but increased resistin expression, causing lipid metabolism disorder in hepatocytes and subsequent dyslipidemia in mice. Although arsenic/ATRA therapy degraded PML-RARα and restored PPARγ expression, it exacerbated dyslipidemia in APL patients. The elevated TRIB3 expression in response to arsenic/ATRA therapy suppressed PPARγ activity by disrupting the PPARγ/RXR dimer, which resulted in dyslipidemia in APL patients undergoing therapy. Indeed, the PPAR activator not only enhanced the anti-APL effects of arsenic/ATRA by suppressing TRIB3 expression but also reduced therapy-induced dyslipidemia in APL patients.

**Conclusion:** Our work reveals the critical role of the PML-RARα/PPARγ/TRIB3 axis in the development of dyslipidemia in APL patients, potentially conferring a rationale for combining ATRA/arsenic with the PPAR activator for APL treatment.

## Introduction

Acute promyelocytic leukemia (APL) is the M3 subtype of acute myelogenous leukemia (AML), which is driven by a chimeric PML-RAPα oncoprotein 1. Although increased body mass index (BMI) and a high prevalence of obesity were reported in patients with APL 2-6, the status of the serum lipid profile in newly diagnosed APL patients remains unclear. All-trans retinoic acid (ATRA) and arsenic trioxide (As_2_O_3_) have long been used successfully against APL 7-9. However, in recent years, more attention has been paid to ATRA-induced hypertriglyceridemia in APL patients undergoing ATRA therapy 10-13. Two mechanisms are assumed to account for the hypertriglyceridemia induced by ATRA. First, ATRA stimulation increases the synthesis of cholesterol and triglycerides in the liver to elevate the blood lipid levels of APL patients 10. Second, metabolites, including cytokines and adipokines, produced by APL cells may contribute to ATRA-induced hypertriglyceridemia 14, 15. However, the molecular mechanism of anti-APL therapy-mediated dyslipidemia remains elusive and needs to be further clarified.

As a nuclear receptor with transcription factor functions, peroxisome proliferator-activated receptor-γ (PPARγ) controls lipid and glucose metabolism by forming PPARγ-retinoid X receptor (RXR) heterodimers to bind a PPAR-response element (PPRE) 16. Retinoic acid receptors (RARs) are ligand-controlled transcription factors that act as heterodimers with RXRs to regulate cell growth and survival and are also implicated in the pathogenesis of metabolic diseases 17. In APL, the oncoprotein PML-RARα can heterodimerize with RXRs, which bind strongly to retinoic acid response elements and represses the transcription of RAR targets 18, 19. Given that crosstalk exists between the nuclear receptors PPARγ, RARs and RXRs and that PML-RARα and PPARγ share the same partner, RXRs, we presumed that PML-RARα contributes to abnormal lipid metabolism by competing with PPARγ for RXR partners to inhibit PPARγ target genes.

Tribbles homologue 3 (TRIB3), a member of the pseudokinase family, acts as a stress sensor that responds to a diverse range of stressors, including inflammatory, metabolic and endoplasmic reticulum (ER) stress 20-22. Our recent study reported that TRIB3 promotes APL progression by interacting with the oncoprotein PML-RARα and inhibiting p53-mediated senescence 23, 24. Increases in TRIB3 expression induced by ATRA or arsenic treatment decreased the therapeutic efficacy of treatment 23. TRIB3 functions as a metabolic stress factor to participate in the regulation of lipid and glucose metabolism by interacting with the E3 ubiquitin ligase COP1, decreasing phospho-AKT and negatively regulating PPARγ transcriptional activity 25-27. Therefore, we herein hypothesize that the increased TRIB3 expression functions together with PML-RARα to participate in the regulation of dyslipidemia in patients with APL. We first conducted a retrospective study to investigate the lipid profiles of an adequate sample of APL patients. Furthermore, we examined the roles and mechanisms of PML-RARα and TRIB3 in lipid metabolism in APL patients before treatment and after induction therapy. Overall, our study not only defines a mechanism by which the crosstalk of PML-RARα/PPARγ/TRIB3 contributes to the abnormal lipid metabolism associated with APL but also provides a rationale for the combination of ATRA/arsenic with PPAR activator for APL therapy.

## Materials and Methods

### Patients and samples

We conducted a retrospective study of 120 patients with AML (APL and non-APL) at our center from January 2014 through June 2016. The eligibility criteria included patients with an age ranging from 15 to 65 years old and newly diagnosed AML. APL patients received ATRA and arsenic, and non-APL patients received idarubicin (10 mg/m^2^/d × 3 days) or daunorubicin (45 mg/m^2^/d × 3 days) and cytarabine (100 mg/m^2^/d × 7 days) as induction therapy. In addition to age, gender, height, weight and hematological parameters, the total cholesterol (TC), triglyceride (TG), high-density lipoprotein (HDL) cholesterol, and low-density lipoprotein (LDL) cholesterol concentrations were measured before and after induction therapy in all patients.

Intravenous blood was collected from all subjects after 10 ± 2 h of fasting to measure serum lipids. Blood samples were collected in vials containing an EDTA anticoagulant agent. The plasma was promptly separated (< 4 h after collection of whole blood). We used an Abbott ARCHITECT c 16000 instrument and TG, TC, HDL, and LDL test kits (Merit Choice Bioengineering (Beijing) Co., Ltd.), which used the GPO-PAP, CHOP-PAP, catalase clearance and surfactant clearance methods, respectively. Abnormal lipid status was determined by utilizing criteria established by the expert panel of the National Cholesterol Education Program (NCEP), Adult Treatment Panel III (ATP III). The cut-off values, including the upper limits of normal, for TGs, TC, and LDL were 1.7 mmol/L (150 mg/dL), 5.2 mmol/L (200 mg/dL), and 3.4 mmol/L (130 mg/dL), respectively, and the lower limit of normal for HDL was 1.04 mmol/L (60 mg/dL). Informed consent was obtained from all participants in accordance with the Declaration of Helsinki. The procedure was approved by the Ethics Committee of the Institute of Hematology and Blood Diseases Hospital of PUMC (KT2019055-EC-1) and the institutional review board at Affiliated Hospital of Xuzhou Medical University. Patient-related information is shown in Supplementary Table 1.

### Definitions of variables

According to the European Society of Cardiology (ESC)/European Atherosclerosis Society (EAS) Guidelines for the Management of Dyslipidemias, hypertriglyceridemia is defined as TGs >1.7 mmol/L (150 mg/dL). For TG-based analysis, the study groups were categorized into two major TG groups: TG group-1 (TG ≤ 1.7 mmol/L) and TG group-2 (TG > 1.7 mmol/L). BMI was calculated as weight in kilograms/(height in meters)^2^, and the current WHO criteria were used to categorize patients as underweight/normal (BMI < 25 kg/m^2^) or overweight/obese (BMI ≥ 25 kg/m^2^). The initial white blood cell (WBC) count was evaluated and adjusted for the APL patients as follows: WBC counts ≤ 10 × 10^9^/L and > 10 × 10^9^/L for the low- and high-risk categories, respectively 28.

### Animal Studies

The myeloid cell-specific *Pml-Rarα* knockin (*Pml-Rarα^KI^*), *Pml-Rarα^KI^ Trib3* knockin *(PR-T3^KI^*), and *Pml-Rarα^KI^ Trib3* knockout *(PR-T3^KO^*) transgenic mice (C57BL/6, male) were constructed as described previously 23. *hMRP8-Pml-Rarα* transgenic mice were obtained from Kan-kan Wang's laboratory 29, 30. These mice were maintained in the animal facility at the Institute of Materia Medica under specific-pathogen-free (SPF) conditions. For the animal studies, the mice were earmarked before grouping and were then randomly separated into groups by one person; however, no particular method of randomization was used. The sample size was predetermined empirically according to previous experience using the same strains and treatments. No animals were excluded from the analysis. Generally, the investigator was not blinded to the group allocation when assessing the outcome. We ensured that the experimental groups were balanced in terms of animal age and weight. All animal studies were approved by the Animal Experimentation Ethics Committee of the Chinese Academy of Medical Sciences (permit no. 002802), and all procedures were conducted in accordance with the guidelines of the Institutional Animal Care and Use Committees of the Chinese Academy of Medical Sciences. The animal study was also conducted in accordance with the Animal Research: Reporting of *In vivo* Experiments (ARRIVE) guidelines.

### Statistical analysis

The Wilcoxon Mann-Whitney test was used to compare the distributions of numerical variables between patients with APL and patients with other types of AML. The associations between qualitative variables were assessed by the χ^2^ test. All statistics were computed using SPSS software, version 22.0. *P* values < 0.05 were considered statistically significant.

## Results

### APL patients have a higher prevalence of dyslipidemia than non-APL AML patients

**Figure 1:**
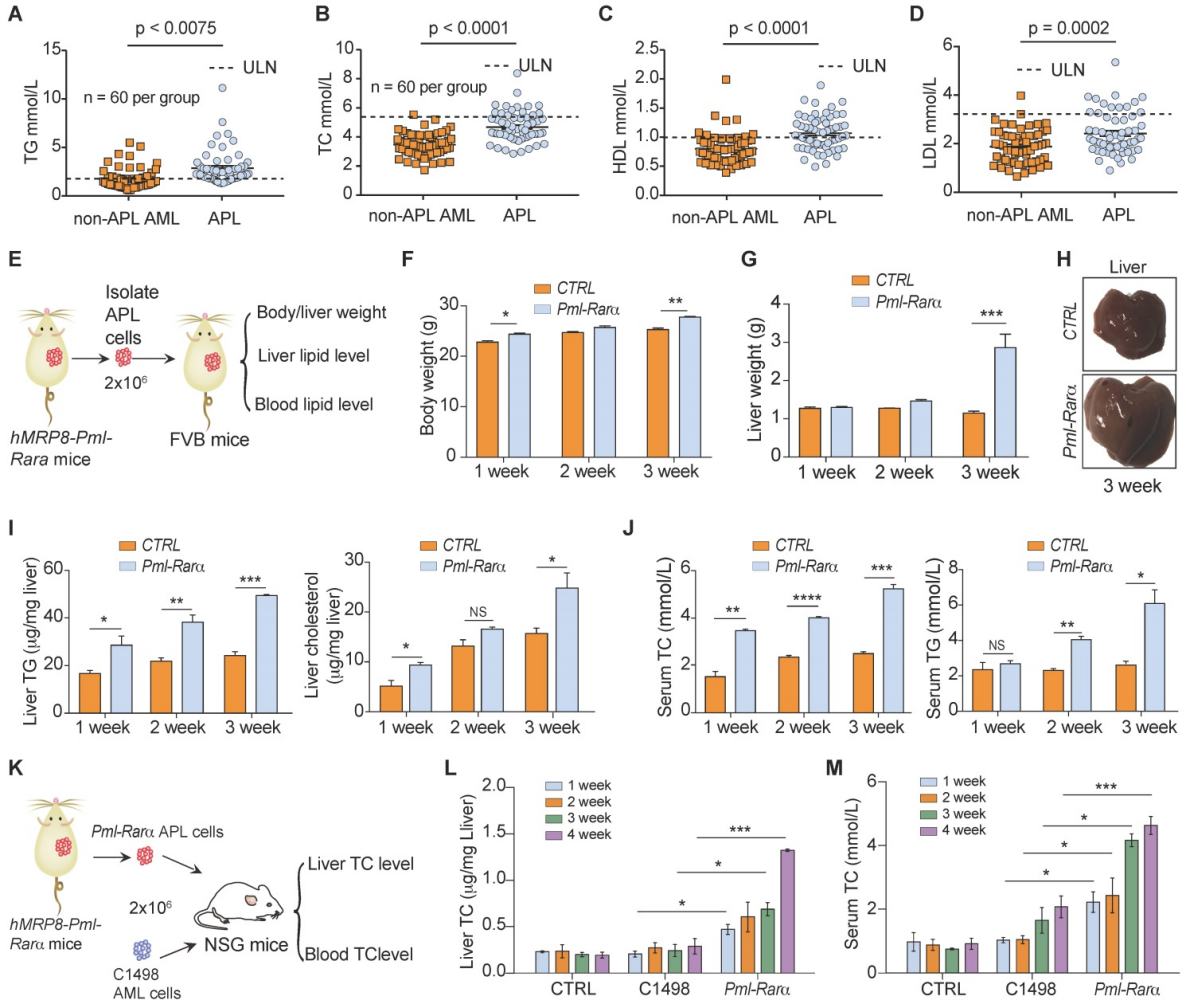
** Lipid profiles of patients with APL and mice transplanted with APL cells from *hMRP8-Pml-Rarα* APL mice. (A-D)** Serum triglyceride (TG), total cholesterol (TC), high-density lipoprotein (HDL) and low-density lipoprotein (LDL) levels in APL patients (n = 60) and non-APL AML patients (n = 60) were evaluated before induction therapy. **(E)** Approaches to define the effects of murine APL cells or normal spleen cells on the body/liver weights, liver lipid levels, and blood lipid levels of FVB recipient mice. **(F and G)** Body weights **(F)** and liver weights **(G)** of the FVB recipient mice transplanted with normal spleen cells (*CTRL*) and *Pml-Rarα* APL cells (*Pml-Rarα*) at the indicated times after inoculation. **(H)** Representative liver morphologies of *CTRL* and *Pml-Rarα* recipient mice at 3 weeks after inoculation. **(I)** Total triglyceride (TG) and cholesterol levels of the liver lipids extracted using the chloroform/methanol method. The data were normalized to the liver weights and are represented as the mean ± the standard error (SEM). N = 3 mice per group.** (J)** Serum TG and total cholesterol (TC) levels in the *CTRL* and *Pml-Rarα* recipient FVB mice at the indicated times after inoculation. **(K)** Approaches to define the effects of murine APL cells or non-APL AML cells (C1498) on liver and blood TC levels in NSG recipient mice. **(L and M)** Liver and serum TC levels in recipient NSG mice transplanted with normal spleen cells (*CTRL*), *Pml-Rarα* APL cells, or C1498 AML cells at the indicated times after inoculation. For panels F-M, n = 4 mice per group. APL: acute promyelocytic leukemia; AML: acute myeloid leukemia; ULN: upper limits of normal.

A retrospective study (Supplementary Table 1) was conducted to investigate lipid profiles and other major clinical parameters in 120 newly diagnosed AML patients (60 APL patients versus 60 non-APL AML patients). More patients were overweight (BMI > 25) in the APL group (52%, 31/60) than in the non-APL AML group (32%, 19/60) (*p* = 0.02). However, the obesity rate (BMI > 30) was not different between the APL and non-APL AML patients (13% vs. 5%, p = 0.11). Hyperlipidemia was found in 65% (39/60) of APL patients and in 36% (22/60) of non-APL patients (*p* = 0.0019). The initial levels of TGs before treatment were higher in the APL patients than in the non-APL patients (Figure 1A). Moreover, the TC, HDL and LDL levels in the APL patients were higher than those in the non-APL patients (Figure 1B-D), indicating that a higher proportion of APL patients had dyslipidemia.

To examine the effect of APL cells on dyslipidemia* in vivo*, normal FVB mice were transplanted with APL cells from *hMRP8-Pml-Rarα* mice, and the body/liver weight, liver lipid levels, and blood lipid levels of recipient mice were assessed over time (Figure 1E). The body and liver weights of mice transplanted with APL cells were higher than those of mice transplanted with normal spleen cells (Figure 1F-H). Additionally, the APL cell-transplanted mice showed elevated TG and cholesterol levels in the liver (Figure 1I). Moreover, the serum TC and TG levels in APL cell-transplanted mice were higher than those in normal spleen cell-transplanted mice (Figure 1J). To explore whether APL cells are the main inducers of dyslipidemia* in vivo,* we assessed the liver and serum TC levels in NOD scid gamma (NSG) mice transplanted with APL or non-APL C1498 AML cells (Figure 1K). Consistent with the clinical data, the liver and serum TC levels in NSG mice transplanted with APL cells were higher than those of C1498 cell-transplanted NSG mice over time after inoculation (Figure 1L-M). Overall, these data indicate that APL cells play a critical role in the enhancement of liver and serum lipid levels in both APL patients and mice.

**Figure 2:**
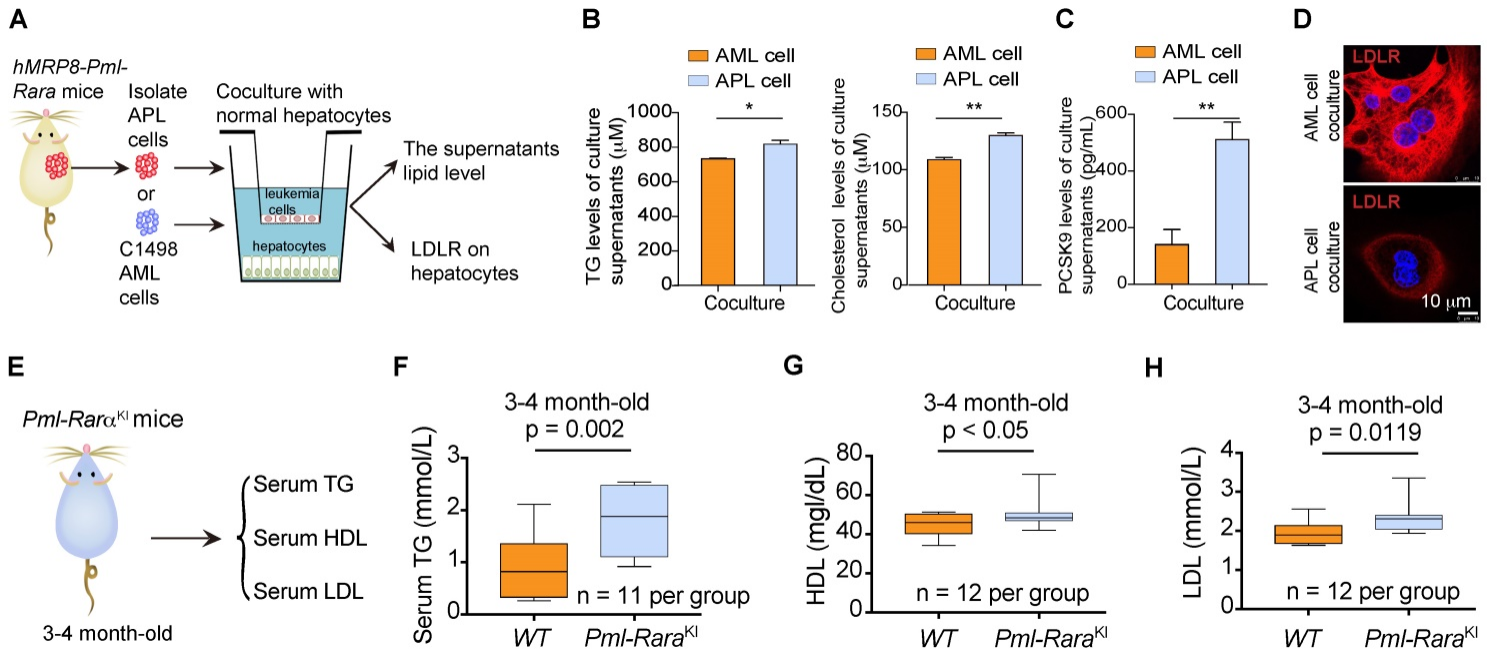
***PML-RARα*-positive APL cells induced lipid production in hepatocytes. (A)** Approaches to define the effects of murine APL cells or C1498 AML cells on lipid production and LDLR expression in hepatocytes. **(B)** Total triglyceride (TG) and cholesterol levels in culture supernatants from mouse hepatocytes were determined by enzymatic assay.** (C)** The PCSK9 levels in the culture supernatants of mouse hepatocytes cocultured with the indicated cells were detected by ELISA. **(D)** Confocal assay of LDLR expression in mouse hepatocytes cocultured with C1498 AML or APL cells. **(E)** Approaches to define murine serum TG/HDL/LDL levels in myeloid cell-specific *Pml-Rarα* knockin (*Pml-Rarα^KI^*) mice (3-4 months old). **(F-H)** Serum TG, HDL, and LDL levels in WT mice and *Pml-Rarα^KI^* mice at the age of 3-4 months.

Given that the lipid production capacity of leukemia cells is less than that of metabolic organs, such as the liver, we next examined the effects of APL cells on the lipid metabolism of hepatocytes (Figure 2A). We found that the triglyceride and cholesterol production in mouse primary hepatocytes was increased by APL cells derived from *hMRP3-Pml-Rara* mice but not by murine non-APL AML cells (Figure 2B). Simultaneously, APL cells promoted the secretion of the proprotein convertase subtilisin/kexin type 9 (PCSK9) in hepatocytes as indicated by ELISA (Figure 2C) and subsequently reduced low-density lipoprotein receptor (LDLR) expression in hepatocytes (Figure 2D). *PML-RARα* acts as the driver of genetic alteration and the most critical factor responsible for the pathogenesis of > 95% of APL cases. To investigate whether PML-RARα contributed to the dyslipidemia associated with APL, we examined the serum TG, HDL and LDL levels in myeloid cell-specific *Pml-Rarα* knockin (*Pml-Rarα*^KI^) mice (Figure 2E). Serum TG, HDL, and LDL levels were higher in 3- to 4-month-old *Pml-Rarα*^KI^ mice (no APL symptoms) than in wild-type (WT) mice of the same age (Figure 2F-H), indicating that *PML-RARα* plays a critical role in the lipid metabolism disorder associated with APL.

### PML-RARα inhibits PPARγ transcriptional activity to induce dyslipidemia in APL patients

**Figure 3:**
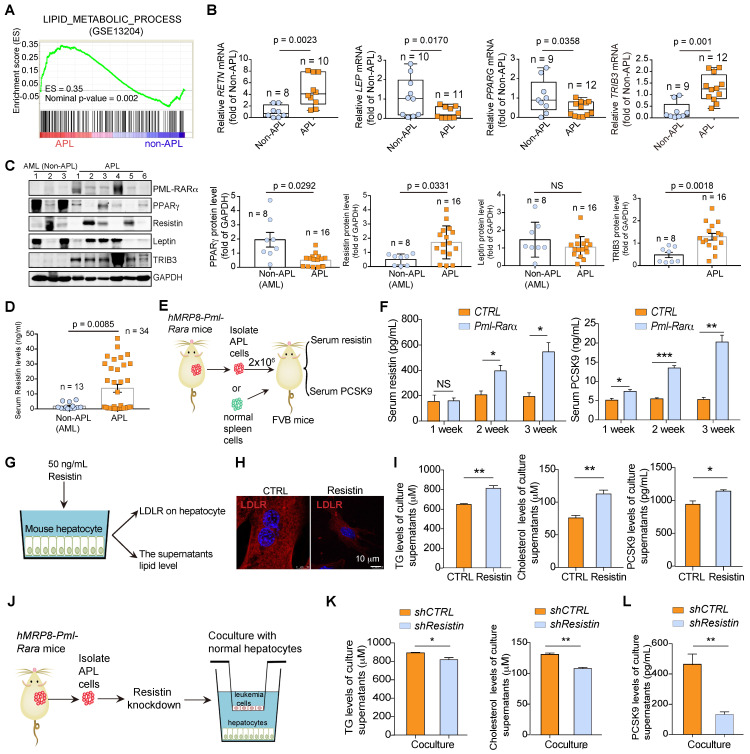
** PPARγ signaling is dysregulated in PML-RARα-positive cells. (A)** Gene set enrichment analysis (GSEA) shows the enrichment of lipid metabolism process-related genes (GSE13204) in APL cells (n = 50) versus non-APL AML cells (n = 50). **(B)** qRT-PCR was performed to analyze the mRNA levels of *RETN*, *LEP, PPARG,* and *TRIB3* (normalized to *GAPDH*) in primary APL cells and non-APL AML cells. **(C left)** The expression of PML-RARα, PPARγ, resistin, leptin, and TRIB3 was detected by Western blotting in primary APL cells and non-APL AML cells.** (C right)** Statistical analyses of PPARγ, resistin, leptin, and TRIB3 expression in primary APL cells (n = 16) and non-APL AML cells (n = 8).** (D)** Serum resistin levels in newly diagnosed APL patients (n = 34) and non-APL AML patients (n = 13). **(E)** Approaches to define the effects of murine APL cells or normal spleen cells on serum resistin and PCSK9 levels in FVB recipient mice. **(F)** Serum resistin (left) and PCSK9 (right) levels in FVB recipient mice transplanted with normal spleen cells (*CTRL*) and APL cells (*Pml-Rarα*) at the indicated times after inoculation (n = 4 per group). **(G)** Approaches to define the effects of resistin on the LDLR expression and lipid secretion of hepatocytes. **(H)** The LDLR expression in normal mouse hepatocytes was detected by confocal assay after resistin (50 ng/mL) stimulation for 24 h.** (I)** The levels of TG, cholesterol and PCSK9 in the supernatants of normal mouse hepatocytes after resistin (50 ng/mL) stimulation for 24 h. (**J**) Approaches to define the effects of resistin-depleted murine APL cells on the TG, cholesterol and PCSK9 secretion of hepatocytes. **(K and L)** The levels of TG, cholesterol and PCSK9 in the supernatants of normal mouse hepatocytes after coculture with APL cells with or without resistin depletion for 24 h.

To investigate the mechanisms underlying the high prevalence of dyslipidemia in APL patients, we performed gene set enrichment analysis (GSEA) on differentially expressed genes (DEGs) enriched in APL and non-APL AML subtypes. Notably, the lipid metabolism process was identified as one of the most significantly enriched Kyoto Encyclopedia of Genes and Genomes (KEGG) gene sets associated with APL (Figure 3A). Moreover, several lipid metabolism-related genes (*RETN*, *GPHN*, *ME1*, *LEP*, *LTC4S*, *DHCR7*, *TRIB3*, *MOSC2*, *ABCA1*, and *PPARG*) ranked among the top dysregulated genes in APL patients versus non-APL AML patients. We further analyzed the individual expression of the lipid metabolism genes in these AML subtypes and found that the expression levels of only *RETN*, *LEP, PPARG* and *TRIB3* were significantly dysregulated in APL cells compared with non-APL AML cells (Figure 3B, Figure S1A and Table S2). Leukemia cells from most APL patients showed reduced PPARγ protein expression and enhanced resistin and TRIB3 protein levels compared with those in leukemia cells from non-APL patients (Figure 3C). The adipokines leptin and resistin, transcriptionally regulated by PPARγ, are involved in the regulation of glucose and lipid metabolism. Serum resistin, but not leptin, was also dysregulated in APL patients (Figure 3D and Figure S1B). Furthermore, overexpression of PML-RARα decreased the protein level of PPARγ in non-APL U937 cells, accompanied by increased resistin and reduced leptin expression (Figure S1C-D). Thus, the serum resistin and PCSK9 levels in APL cell-transplanted FVB mice were higher than those in the control mice over time (Figure 3E-F). To verify whether the adipokine resistin secreted from APL cells could enhance lipid levels in hepatocytes, we treated normal liver cells with resistin (Figure 3G) and found that resistin stimulation lowered LDLR expression in hepatocytes and enhanced the levels of TG, TC and PCSK9 in the culture supernatants of hepatocytes (Figure 3H-I). Consistently, *resistin*-knockdown APL cells almost lost the ability to enhance the levels of TG, TC and PCSK9 in the culture supernatants of hepatocytes (Figure 3J-L). Collectively, these data indicate that APL cells elevate lipid levels in APL mice and that PML-RARα acts as the core factor causing the dyslipidemia associated with APL.

**Figure 4:**
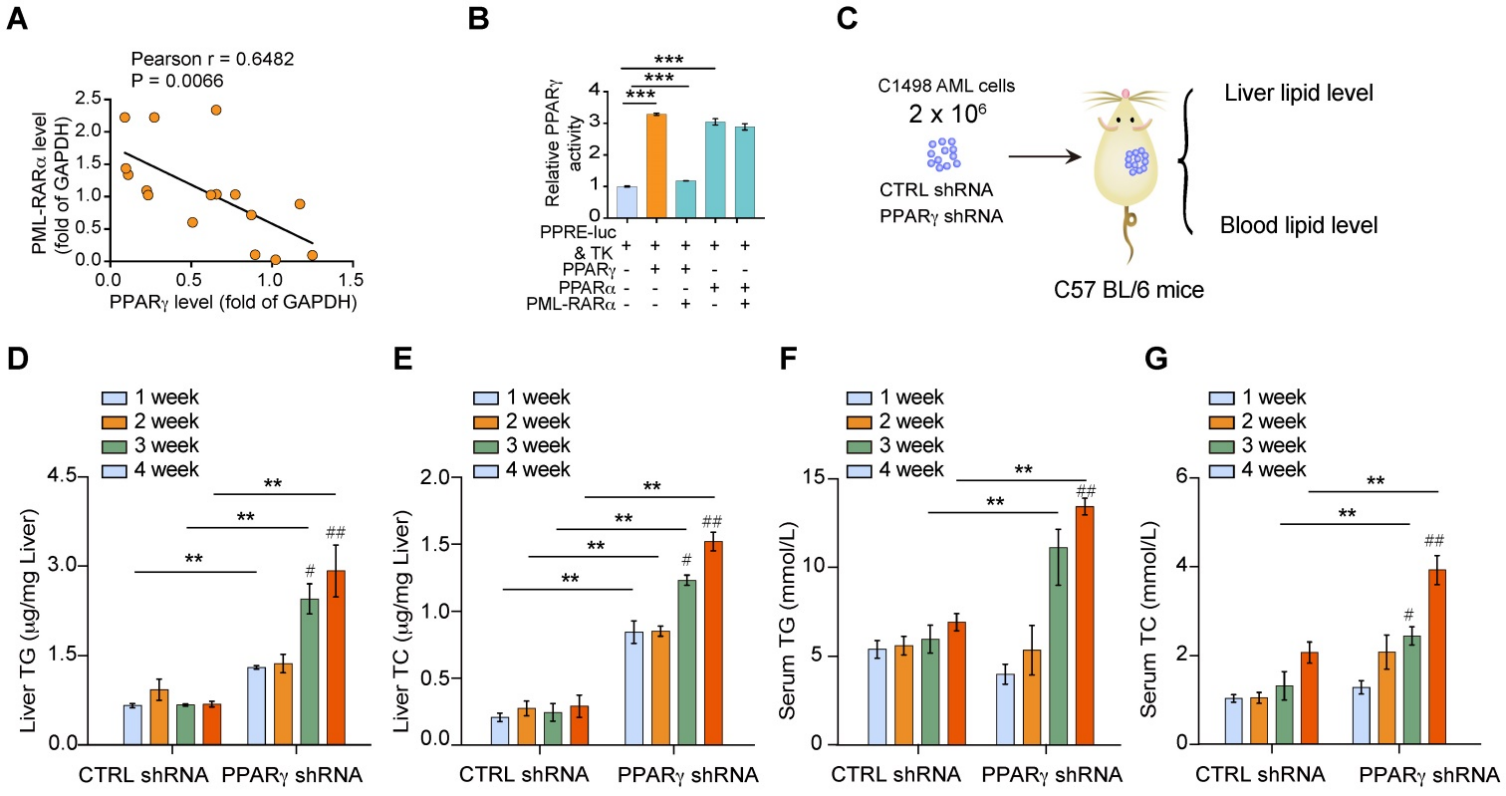
** Reduced PPAR**γ** activity in APL cells causes lipid metabolism disorder in APL patients. (A)** Correlation between PML-RARα and PPARγ expression (protein level) in human primary APL cells (n = 16). Each data point represents the value from an individual patient. Statistical significance was measured by Pearson's correlation test. **(B)** PML-RARα decreased the transcriptional activity of PPARγ. HEK 293T cells were transiently transfected with the indicated plasmids. After 24 h of transfection, luciferase activities were measured.** (C)** Approaches to define the effects of C1498 AML cells with or without *PPARγ* depletion on the liver and blood lipid levels of C57 BL/6 recipient mice. **(D-G)** Liver and serum TG and TC levels in mice transplanted with CTRL shRNA C1498 cells or PPARγ shRNA C1498 cells at the indicated times after inoculation. For panel D-G, n = 4 mice per group.

To determine how PML-RARα is involved in the metabolic disorders associated with APL, we examined the expression of PM-RARα and PPARγ in leukemia cells from APL patients. The expression of PML-RARα was negatively correlated with that of PPARγ in APL cells (Figure 4A). The transcriptional activity of PPARγ, but not PPARα, was inhibited by overexpression of PML-RARα in APL cells (Figure 4B). Indeed, adipose PPARγ is a well-known mediator of organism-wide metabolism 16; however, it is still unclear whether myeloid PPARγ has similar effects. Given that APL cells have a low level of endogenous PPARγ protein, PPARγ expression was stably depleted in non-APL myeloid leukemia cells, and the effects of these cells on the liver and serum lipid levels were examined in the mice (Figure 4C). Non-APL myeloid leukemia cells induced no liver TG or TC enhancement over time *in vivo*, but *PPAR*γ knockdown cells increased both the liver TG and TC levels of the transplanted mice (Figure 4D & E). Similarly, increases in serum TG and TC levels were observed in mice transplanted with *PPAR*γ-depleted leukemia cells but not in mice transplanted with control leukemia cells (Figure 4D & E). Moreover, the lipid levels of mice transplanted with *PPAR*γ-depleted leukemia cells were much higher than those of control mice (Figure 4D-G). These data indicate that altered PPARγ activity in APL cells is responsible for the dyslipidemia observed in APL mice.

**Figure 5:**
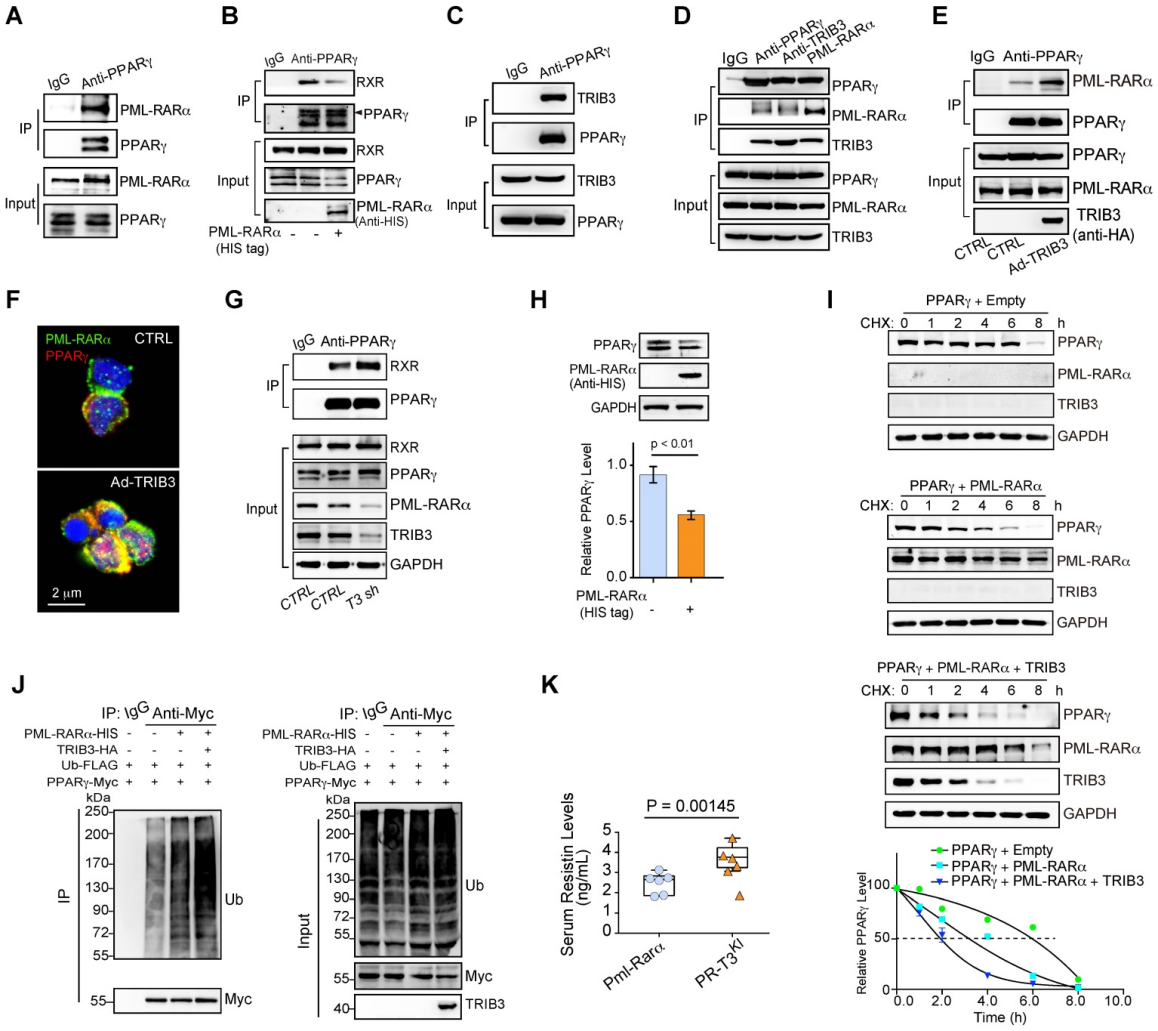
** The combination of PML-RARα and TRIB3 inhibits PPARγ activity by disrupting the PPARγ/RXR heterodimer and promoting PPARγ degradation. (A)** The interaction between endogenous PML-RARα and PPARγ was detected by a coimmunoprecipitation (co-IP) assay in human primary APL cells. **(B)** PML-RARα reduced the interaction between PPARγ and RXR. NB4 cells were transfected with or without a PML-RARα-expressing plasmid. After 24 h of transfection, co-IP analysis was performed to detect the PPARγ/RXR interaction. **(C)** The interaction between TRIB3 and PPARγ was detected by a co-IP assay in HEK 293T cells transfected with TRIB3- and PPARγ-expressing plasmids. **(D)** TRIB3, PML-RARα and PPARγ trimers were detected by a co-IP assay in HEK 293T cells. HEK 293T cells were transiently transfected with PPARγ-, TRIB3-, and PML-RARα-expressing plasmids. After 24 h of transfection, a co-IP assay was performed to detect the interactions between TRIB3, PML-RARα and PPARγ. **(E)** TRIB3 increased the interaction between PPARγ and PML-RARα. NB4 cells were infected with an adenovirus expressing TRIB3 or an HA tag (CTRL). After 24 h of transfection, a co-IP analysis was performed to detect the PPARγ/PML-RARα interaction. **(F)** TRIB3 increased the colocalization of PPARγ and PML-RARα. Human primary APL cells were infected with adenovirus expressing TRIB3 or CTRL. After 24 h of transfection, the colocalization of PPARγ/PML-RARα was detected by an IF staining assay. **(G)** *TRIB3* depletion increased the interaction between PPARγ and RXR. A co-IP analysis was performed to detect the PPARγ/RXR interaction in NB4 cells with or without *TRIB3* depletion. **(H)** The effects of PML-RARα on the protein level of PPARγ in U937 cells. **(I)** The effect of PML-RARα or PML-RARα and TRIB3 overexpression on PPARγ degradation. HEK 293T cells were transfected with the indicated plasmids, and 12 h later, the cells were incubated with CHX (10 μg/mL) for the indicated times. **(J)** The effect of PML-RARα overexpression or PML-RARα and TRIB3 overexpression on PPARγ ubiquitination. HEK 293T cells were transfected with the indicated plasmids, and 12 h later, cell extracts were IP with anti-Myc Ab. Ubiquitinated PPARγ was detected by immunoblotting. **(K)** Serum resistin levels in *Pml-Rarα* mice (nonleukemic) with or without *Trib3* knockin (3 months old).

PPARγ is a ligand-activated transcription factor and functions as a heterodimer with an RXR 31. Mechanistically, this finding was verified by the interaction of PML-RARα and PPARγ in human APL cells (Figure 5A). PML-RARα overexpression decreased the interaction of PPARγ and RXR in APL cells (Figure 5B). Elevated TRIB3 expression promotes lipid metabolism and sustains the oncogenic function of PML-RARα in APL patients via protein-protein interactions 23. Indeed, TRIB3 coimmunoprecipitated with PPARγ in APL cells (Figure 5C). Furthermore, TRIB3, PPARγ and PML-RARα formed a heterotrimer (Figure 5D), and elevated TRIB3 promoted the binding of PML-RARα and PPARγ in APL cells (Figure 5E-F). Moreover, *TRIB3* depletion rescued the interaction between PPARγ and RXR by decreasing PML-RARα expression (Figure 5G). These data indicate that the collaboration of PML-RARα and TRIB3 inhibits PPARγ activity by disrupting the PPARγ/RXR heterodimer.

Additionally, overexpression of PML-RARα decreased PPARγ protein expression (Figure 5H). We assessed the role of PML-RARα in the regulation of PPARγ ubiquitination and degradation mediated by ubiquitin ligases 32, 33. In the presence of the protein synthesis inhibitor cycloheximide (CHX), the PPARγ protein exhibited a reduced half-life in PML-RARα-overexpressing cells compared with that in the control group. *TRIB3* overexpression further accelerated the degradation of PPARγ mediated by PML-RARα overexpression (Figure 5I). PML-RARα overexpression increased the ubiquitination of PPARγ and TRIB3 further increased the ubiquitination of PPARγ mediated by PML-RARα overexpression (Figure 5J). Furthermore, *Trib3* knockin *Pml-Rarα (PR-T3^KI^)* mice 23 showed elevated serum resistin levels compared with those in the *Pml-Rarα^KI^*mice (Figure 5K). Given the interconnectivity between PML-RARA expression and PPARG/TRIB3, we investigated whether these genes are direct targets (transcriptionally) of PML-RARα by transfecting Myc-tagged PML-RARA plasmids into NB4 cells and used an anti-Myc antibody to capture protein-DNA complexes. The ChIP-qPCR results verified that both *PPARG* and *TRIB3* are direct target genes of PML-RARA (Figure S2). Thus, these gain-of-function studies indicate crucial roles of PML-RARα and TRIB3 in the inhibition of PPARγ activity by interrupting the PPARγ/RXR heterodimer and promoting ubiquitination-dependent PPARγ degradation.

### ATRA/arsenic rescues PPARγ expression in APL cells but does not ameliorate dyslipidemia in APL patients

**Figure 6:**
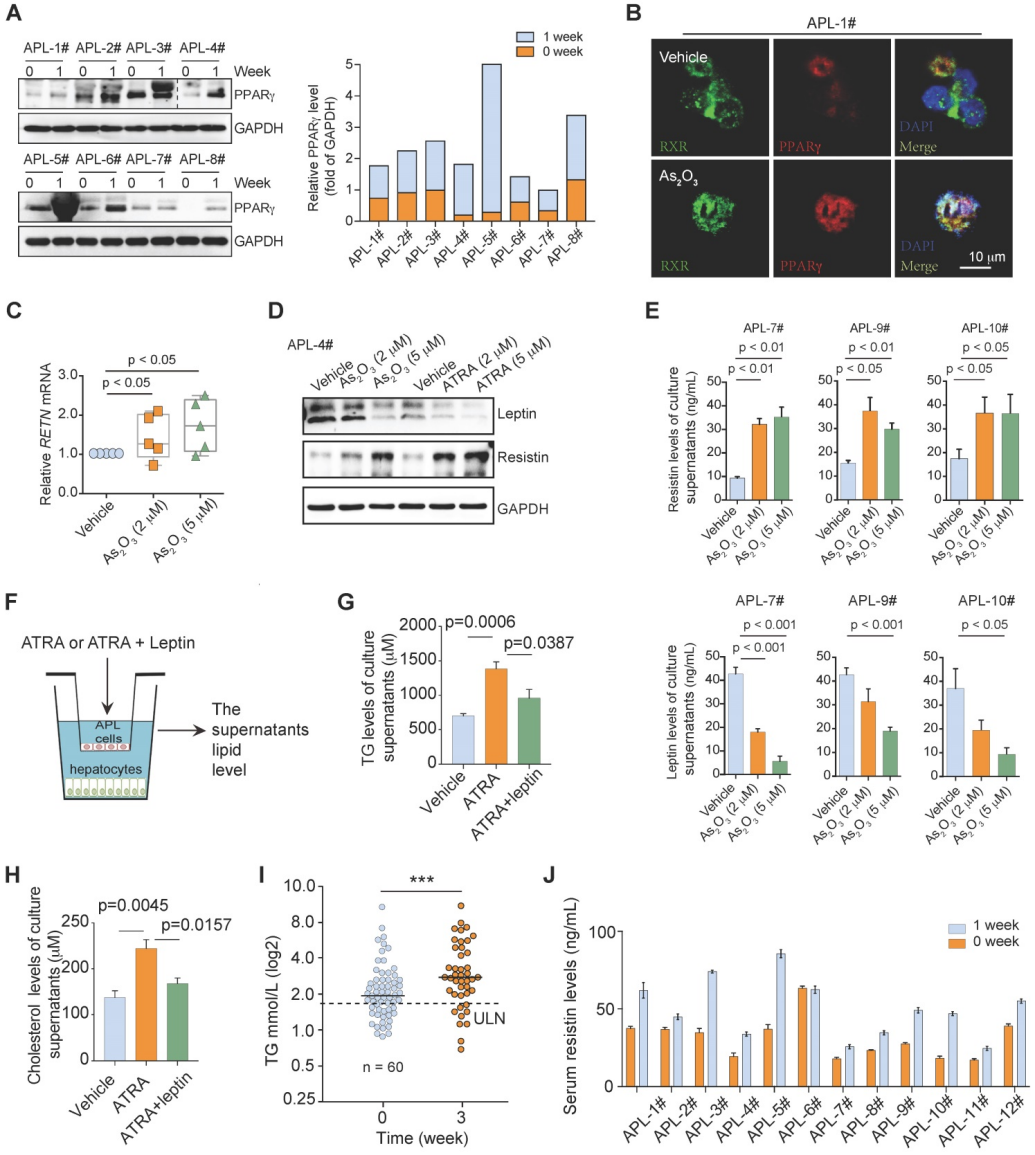
** ATRA/As_2_O_3_ treatment increases PPARγ expression but does not improve dyslipidemia. (A)** ATRA/As_2_O_3_ treatment increased PPARγ expression in APL cells. The PPARγ expression in primary APL cells isolated from the bone marrow of APL patients was detected by Western blotting before treatment and 1 week after combined ATRA and arsenic treatment.** (B)** Colocalization of PPARγ (red) and RXR (green) in primary human APL cells (APL-1#) was detected by immunostaining. Scale bar, 10 μm.** (C)** qRT-PCR was performed to analyze the *RETN* mRNA levels in primary APL cells treated as indicated for 24 h.** (D)** The expression of leptin and resistin in primary APL cells was detected by Western blotting after administration of the indicated treatment for 24 h.** (E)** Effects of arsenic treatment on resistin and leptin secretion in primary APL cells after 2 days of treatment. **(F)** Approaches to define the effects of ATRA or ATRA and leptin on the lipid production of murine hepatocytes cocultured with murine APL cells. **(G and H)** The levels of TG **(G)** and cholesterol **(H)** in the supernatants of normal mouse hepatocytes after coculture with primary APL cells treated with ATRA or ATRA and leptin for 12 h. **(I)** ATRA and arsenic treatment increased serum TG levels in APL patients. The serum TG levels of APL patients were measured by Abbott ARCHITECT c16000 at the indicated treatment times (n = 60), ULN: upper limits of normal.** (J)** ATRA and arsenic treatment increased serum resistin levels in APL patients (n = 12).

ATRA plus arsenic trioxide (As_2_O_3_) with or without chemotherapy induces high remission rates in APL patients by degrading the oncoprotein PML-RARα 34, 35. Given that PML-RARα inhibited PPARγ activity in APL cells, we next examined whether ATRA/As_2_O_3_ rescued PPARγ activity and improved dyslipidemia in APL patients by decreasing PML-RARα expression. The combination of ATRA and As_2_O_3_ enhanced PPARγ expression in leukemia cells from APL patients after 1 week of induction therapy (Figure 6A). Furthermore, As_2_O_3_ treatment rescued the colocalization of PPARγ and RXR by degrading the PML-RARα protein (Figure 6B). However, the mRNA and protein levels of resistin were enhanced, whereas the protein level of leptin was decreased in APL cells after As_2_O_3_ or ATRA treatment (Figure 6C & D), which was accompanied by elevated resistin secretion and downregulated leptin secretion (Figure 6E) in the culture supernatant of APL cells after As_2_O_3_ treatment.

We next assessed the effects of leptin on the secreted TC and TG levels of the supernatant of hepatocytes cells cocultured with APL cells treated with ATRA (Figure 6F). Increased TC and TG levels were observed in the ATRA-treated coculture system of primary mouse APL cells and hepatocytes. Leptin partially protected against the ATRA-induced TC and TG enhancement of hepatocytes cocultured with APL cells (Figure 6G & H). These data indicated that decreased leptin contributed to the dyslipidemia phenotypes caused by ATRA treatment. We further analyzed the lipid profiles of APL patients during first-line therapy. The TG concentrations of APL patients increased after 3 weeks of induction therapy (Figure 6I). Consistently, the serum resistin levels of most APL patients were also elevated after 1 week of induction therapy (Figure 6J and Table S2). The observation that anti-APL therapy reduced PML-RARα and enhanced PPARγ but could not normalize dyslipidemia in APL patients indicates that other important regulator(s) may participate in the regulation of lipid metabolism disorder in APL patients during induction therapy.

### High TRIB3 mediates ATRA/arsenic therapy-induced dyslipidemia in APL patients

**Figure 7:**
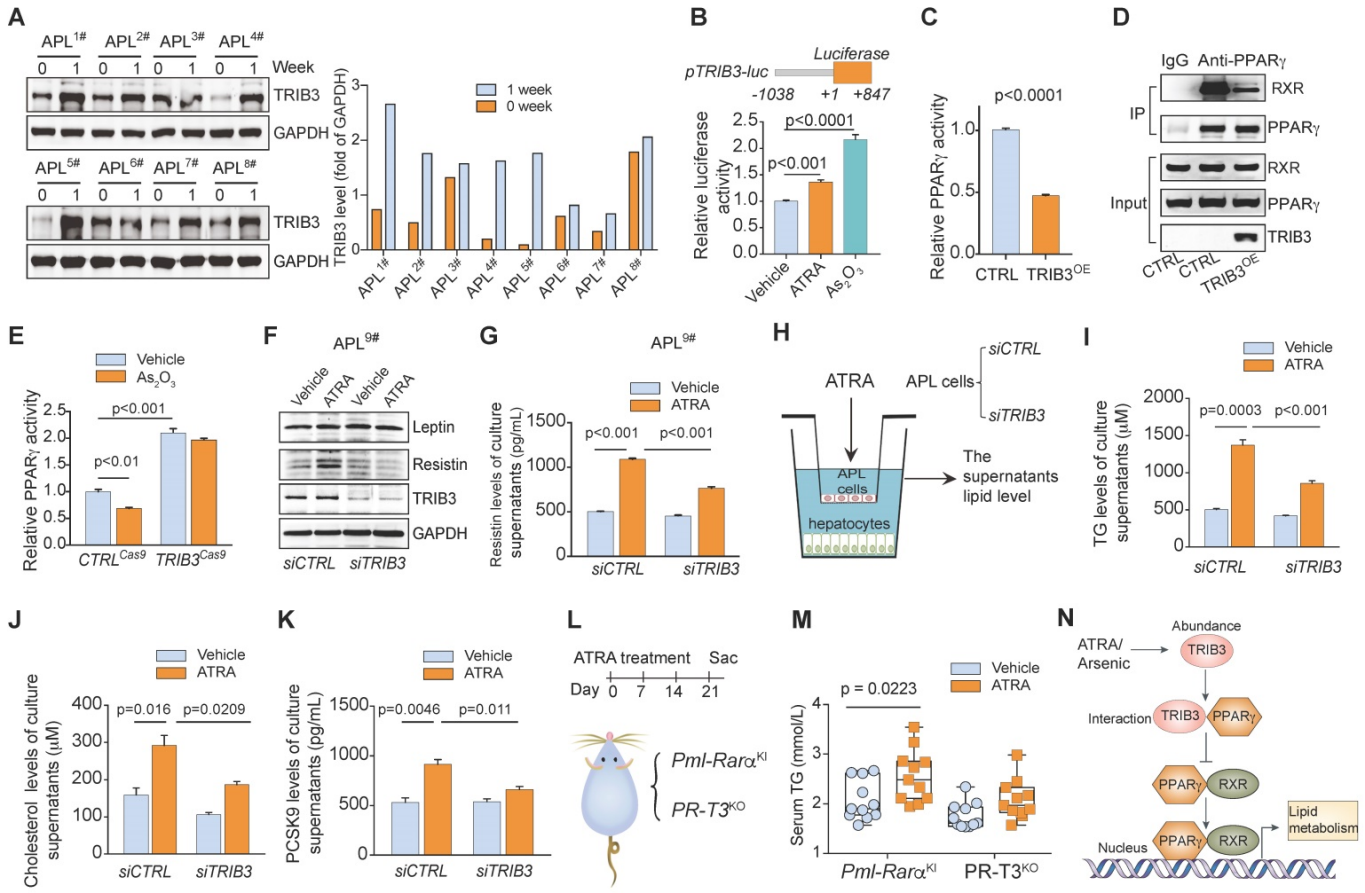
** ATRA/As_2_O_3_-enhanced TRIB3 inhibits PPARγ activity and increases dyslipidemia in APL. (A)** ATRA/As_2_O_3_ treatment induced TRIB3 abundance in APL cells. TRIB3 expression in primary APL cells isolated from the bone marrow of APL patients (n = 8) was detected by Western blotting before treatment and 1 week after combined ATRA and arsenic treatment.** (B)** ATRA/arsenic treatment inhibited *TRIB3* transcription. HEK 293T cells were transiently transfected with the pTRIB3-luc and TK plasmids. After 24 h of transfection, cells were treated with ATRA or arsenic for 24 h, and luciferase activities were measured.** (C)** TRIB3 decreased the transcriptional activity of PPARγ. HEK 293T cells were transiently transfected with PPRE-Luc, TK, and CTRL or TRIB3 overexpression plasmids. After 24 h of transfection, the cells were treated with PZD (5 μM), and luciferase activities were measured. **(D)** TRIB3 reduced the interaction between PPARγ and RXR. NB4 cells were transfected with or without a TRIB3-expressing plasmid. After 24 h of transfection, co-IP analysis was performed to detect the PPARγ/RXR interaction. **(E)** The effect of As_2_O_3_ treatment on the transcriptional activity of PPARγ in NB4 cells with or without *TRIB3* deletion. *CTRL*^Cas9^ and *TRIB3*^cas9^ NB4 cells were transfected with PPARγ reporter genes. After 24 h of transfection, the cells were treated with vehicle or As_2_O_3_ for 24 h, and luciferase activities were measured. **(F)** The effect of ATRA treatment on the protein levels of leptin and resistin in primary APL cells with or without *TRIB3* depletion. **(G)** The effect of ATRA treatment on the resistin level of culture supernatants of primary APL cells with or without *TRIB3* depletion. **(H)** Approaches to define the effects of *TRIB3*-depleted murine APL cells on the TG, cholesterol and PCSK9 secretion of hepatocytes. **(I-K)** The levels of TG **(I)**, cholesterol **(J)** and PCSK9 **(K)** in the supernatants of normal mouse hepatocytes after coculture with *TRIB3*-depleted APL cells treated with ATRA for 12 h.** (L)** Strategy for studying the effects of ATRA treatment on the serum TG levels in *Pml-Rarα* (*Pml-Rarα^KI^*) and *Trib3* knockout *Pml-Rarα* (*PR-T3^KO^*) mice. **(M)** Serum TG levels in the indicated mice treated with or without ATRA (8 months old). **(N)** Schematic diagram illustrating the role of TRIB3 in the regulation of PPARγ activity in APL cells treated with ATRA/arsenic.

We found that combined ATRA and As_2_O_3_ therapy increased TRIB3 abundance in APL cells from most patients after 1 week of therapy (Figure 7A and Table S2). Furthermore, ATRA or As_2_O_3_ enhanced the transcription of TRIB3 in APL cells (Figure 7B). TRIB3 was reported to suppress adipocyte differentiation by negatively regulating PPARγ transcriptional activity 26. Indeed, TRIB3 overexpression inhibited the transcriptional activity of PPARγ (Figure 7C) by interrupting the interaction between RXR and PPARγ (Figure 7D) in APL cells. *TRIB3* deletion not only increased the transcriptional activity of PPARγ but also rescued the reduced PPARγ activity induced by As_2_O_3_ treatment (Figure 7E). Although *TRIB3* depletion showed no effects on leptin expression in APL cells, silencing *TRIB3* impeded the enhancement of resistin expression induced by ATRA treatment in APL cells (Figure 7F). Moreover, *TRIB3* depletion ameliorated the elevated resistin production in the culture supernatants of APL cells treated with ATRA (Figure 7G). Additionally, we examined the effects of ATRA on the levels of TC, TG and PCSK9 in the supernatant of primary hepatocytes cocultured with mouse APL cells with or without *TRIB3* depletion (Figure 7H). Loss of TRIB3 moderately counteracted the increased levels of TC, TG and PCSK9 secretion from hepatocytes cocultured with APL cells treated with ATRA (Figure 7I-K). Importantly, ATRA treatment induced increased serum TG levels in *Pml-Rarα* transgenic mice but did not elevate TG levels in *Trib3-*knockout* Pml-Rarα* transgenic mice (*PR-T3^KO^*) (Figure 7L & M), indicating that TRIB3 is involved in the regulation of anti-APL-therapy-induced dyslipidemia in individuals with APL. Overall, arsenic/ATRA treatment enhances TRIB3 abundance in APL cells, and TRIB3 interacts with PPARγ to impede the heterodimer formation of PPARγ and RXR, inhibiting PPARγ transcriptional activity and abnormal lipid metabolism in APL cells (Figure 7N).

### Collaboration of PPAR agonists and ATRA/arsenic improves dyslipidemia in APL patients

**Figure 8:**
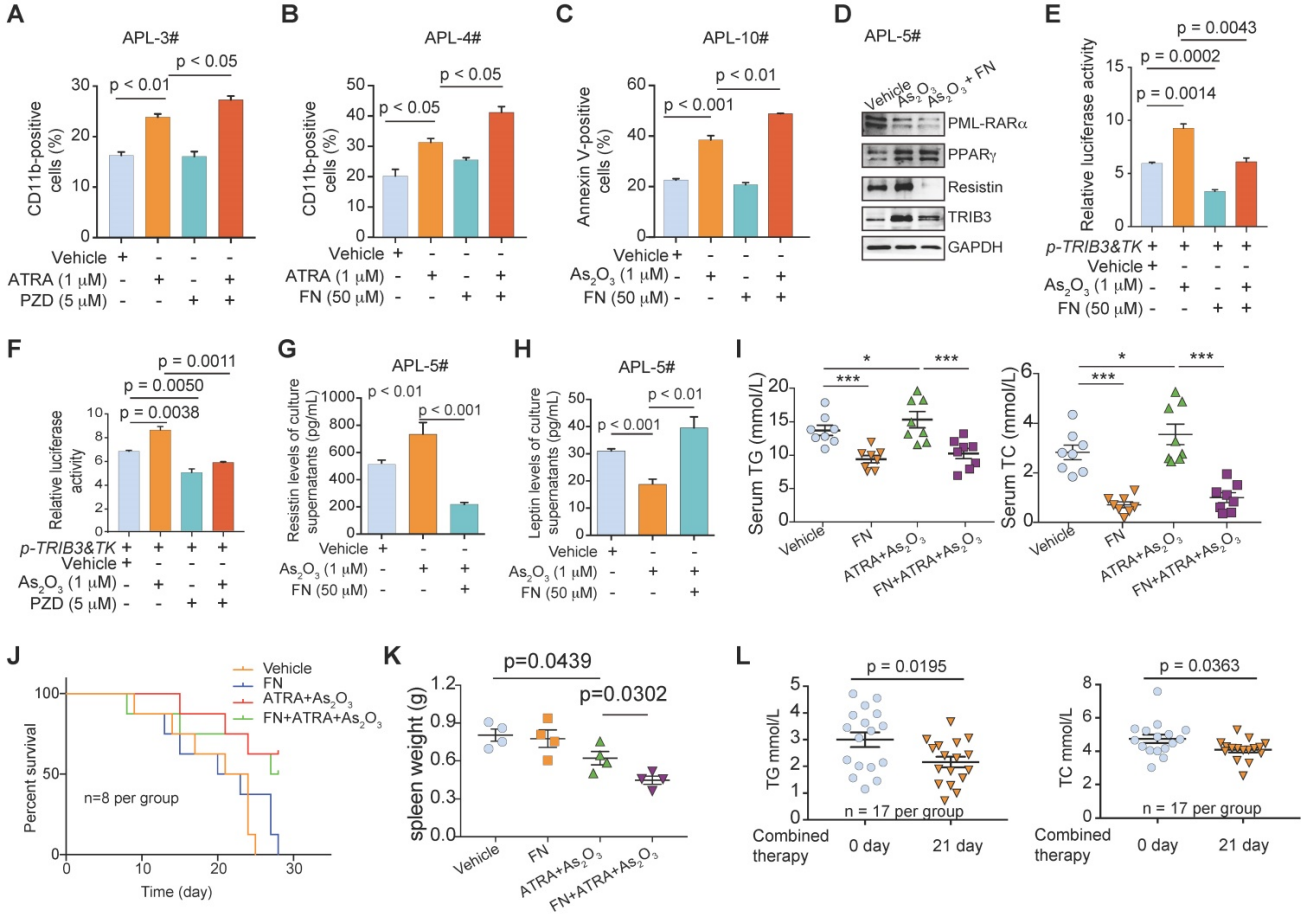
** PPAR agonists induce the synergism of anti-APL with ATRA/As_2_O_3_ by improving dyslipidemia. (A)** CD11b expression was evaluated by flow cytometry in primary APL cells that were subjected to the indicated treatment. The percentage of CD11b-positive cells was calculated with FCS Express software. The data are presented as the mean ± SEM of 3 assays. **(B and C)** The PPARα agonist (fenofibrate, FN) increased the percentage of differentiated and apoptotic APL cells induced by ATRA or As_2_O_3_ treatment. Primary APL cells were treated with the indicated treatment for 24 h and then stained with a CD11b antibody or Annexin-V‑PI and evaluated by flow cytometry. The data are presented as the mean ± SEM of 3 assays.** (D)** The expression of PML-RARα, PPARγ, TRIB3, and resistin in primary APL cells treated as indicated was detected by Western blotting. **(E and F)** PPAR agonists suppressed the elevated TRIB3 transcription induced by As_2_O_3_. HEK 293T cells were transiently transfected with the TRIB3 reporter plasmids (*p-TRIB3&TK*). After 24 h of transfection, the cells were treated with the indicated treatment for 24 h, and luciferase activities were then measured.** (G and H)** The PPARα agonist (FN) enhanced the secreted leptin level and reduced the resistin level in primary APL cells that were subjected to As_2_O_3_ treatment. The data are presented as the mean ± SEM of 3 assays. **(I)** Serum TG and TC levels in *Pml-Rarα* recipient FVB mice treated as indicated.** (J)** Kaplan-Meier survival curves for the *Pml-Rarα* recipient FVB mice treated as indicated (n = 8). **(K)** The data indicate the spleen weights of the *Pml-Rarα* recipient FVB mice treated as indicated. **(L)** Serum TG and TC levels in APL patients (n = 17) were evaluated after combined treatment with ATRA/arsenic plus FN.

Given that APL patients had a high prevalence of dyslipidemia before and during induction therapy, the use of a lipid-lowering drug combined with ATRA and As_2_O_3_ therapy may further benefit APL patients and improve their clinical outcomes. First, we examined the effects of pharmacological PPAR activation in APL cells. A PPARγ activator (pioglitazone, PZD) synergized with ATRA-mediated differentiation in primary APL cells (Figure 8A). Similarly, the PPARα agonist fenofibrate (FN) potently increased the differentiation and apoptosis percentages of APL cells induced by ATRA or an arsenic agent (Figure 8B & C). Furthermore, the PPAR activator decreased PML-RARα expression and impeded the increased resistin induced by As_2_O_3_ treatment (Figure 8D). Interestingly, the arsenic-induced increase in TRIB3 expression was hindered by FN treatment (Figure 8D), and the PPAR activators reduced the increase in TRIB3 transcription induced by arsenic treatment, indicating that PPAR activators nonspecifically inhibit TRIB3 (Figure 8E & F). Accordingly, FN treatment protected against elevated resistin secretion and reduced leptin secretion in APL cells treated with As_2_O_3_ (Figure 8G & H).

We next evaluated the therapeutic effect of ATRA/arsenic and FN in combination in the PML-RARα mouse model. Treatment of PML-RARα APL mice with the combination for 3 weeks improved lipid metabolism disorder associated with APL, as indicated by reductions in the elevated serum TG and TC levels in ATRA/arsenic-treated APL mice (Figure 8I). Although the combination did not significantly improve the survival rate of APL mice compared with those treated with ATRA/arsenic alone (Figure 8J), the PPAR agonist FN synergized with ATRA/arsenic therapy to decrease spleen weights in APL mice (Figure 8K). Similarly, treatment of APL patients (n = 17) with the combination for 3 weeks improved the dyslipidemia associated with APL, as indicated by reductions in the elevated TG and TC levels of APL patients (Figure 8L). Overall, these data showed that combined therapy with ATRA/arsenic and a PPAR agonist is suggested for APL patients.

In summary, our study suggests that the collaboration of PML-RARα with elevated TRIB3 expression inhibits PPARγ activity and causes lipid metabolism abnormalities in newly diagnosed APL patients. The TRIB3 expression that was increased by ATRA/As_2_O_3_ treatment further impeded PPARγ activity by forming the heterotrimer of TRIB3, PML-RARα and PPARγ, contributing to dyslipidemia in APL patients undergoing anti-APL therapy.

## Discussion

Previous studies demonstrated that TRIB3 interaction with PPARγ negatively regulates PPARγ activity in adipose tissue 26. Our recent work indicates that elevated TRIB3 expression stabilizes the oncoproteins PML-RARα and PML in APL 23, 24 and that PML exerts its essential role in breast cancer cell and hepatic stellate cell (HSC) maintenance through regulation of PPAR signaling and fatty acid oxidation (FAO) 36, 37. However, we do not know whether or how TRIB3, PPARγ and PML-RARα contribute collaboratively to the regulation of lipid metabolism in APL cells. In this study, we verified the hypothesis that the metabolic stress sensor TRIB3 collaborates with the oncoprotein PML-RARα to inhibit PPARγ activity and subsequently cause dyslipidemia in newly diagnosed APL patients. Moreover, elevated TRIB3 expression in response to arsenic/ATRA therapy further suppressed PPARγ activity by disrupting the PPARγ/RXR dimer, which contributes to abnormal lipid metabolism in arsenic/ATRA-treated APL patients. Accordingly, the PPAR activator not only enhanced the anti-APL effects of arsenic/ATRA *in vitro* but also reduced arsenic/ATRA-induced dyslipidemia in APL patients. Thus, our study may provide a rationale for the combination of ATRA/arsenic therapy with the PPAR activator for the treatment of patients with APL.

Several studies have shown that APL patients have a higher percentage of obesity 38, which is considered an adverse prognostic indicator for clinical outcome in APL. However, the mechanism of the association between APL and obesity or overweight remains unclear. Less is known regarding the lipid profile statuses of newly diagnosed APL patients, although dyslipidemia has been associated with several types of cancer 39-41. In this study, we showed that newly diagnosed APL patients indeed had a higher prevalence of dyslipidemia than non-APL AML patients, indicated by elevated serum levels of TC, TG, HDL and LDL. PPARγ is a transcription factor that plays a key role in adipogenesis and insulin sensitization, and polymorphisms in PPARγ have been associated with obesity and diabetes-related phenotypes, such as hyperinsulinemia and dyslipidemia 42, 43. Here, we identified that APL cells with defective PPARγ function is likely the essential factor triggering the dyslipidemia of APL patients by secreting resistin and subsequently disrupting the lipid metabolism of hepatocytes. Although Jansen *et al* provided a preliminary clue that PML-RARα interferes with PPAR signaling pathways 19, little is known regarding the role and mechanism of PML-RARα in the lipid metabolism modulated by PPARγ. Based on these observations, our study revealed that PML-RARα interacts with PPARγ to disrupt the PPARγ/RXR heterodimer and promote PPARγ ubiquitination and degradation, which may partially explain the obesity and dyslipidemia in newly diagnosed patients with APL that is driven by PML-RARα. A recent study reported that the cytokine galectin-12, a negative regulator of lipolysis, is selectively overexpressed in APL cells 44 and may participate in lipid metabolism regulation in APL patients via lipid droplet accumulation. Our study showed that the production and secretion of the adipokines resistin and leptin were dysregulated in APL cells. Thus, galectin-12, resistin, leptin, and other adipokines/cytokines regulated by PPARγ may act synergistically to affect lipid metabolism in metabolic tissues, contributing to the dyslipidemia associated with APL.

The application of ATRA/arsenic treatment in APL makes APL a curable chronic disease, a great victory in the war against cancer in humans. However, although ATRA/arsenic treatment degraded PML-RARα and restored PPARγ expression, it did not improve but rather exacerbated dyslipidemia in these APL patients. One major reason is that retinoids induce hypertriglyceridemia in APL patients treated with ATRA. Several studies have indicated that the increased synthesis of cholesterol and TGs, the disproportion of apoprotein constituents in the liver, and defects in very-low-density lipoprotein (VLDL) clearance in skeletal muscle contribute to ATRA-induced hypertriglyceridemia in APL 45-47. However, the detailed molecular mechanism of therapy-related dyslipidemia in APL has not been clear until now. Pseudokinase TRIB3, a critical modulator of glucose/lipid metabolism and cancer progression 48-50, has been reported to inhibit PPARγ activity in adipocytes via its interaction with PPARγ 26. Although we previously found that TRIB3 expression is enhanced in APL cells 23 and arsenic enhances the mRNA and protein expression of TRIB3 51, it is still unclear whether TRIB3 plays a role in the abnormal lipid level of treated APL patients. Our study revealed that TRIB3 induced by ATRA/arsenic therapy further inhibits PPARγ activity to dysregulate TG and adipocytokine secretion in APL cells, which subsequently contributes to the disordered lipid metabolism associated with APL. Thus, the inhibition of TRIB3 combined with ATRA/arsenic treatment may improve the lipid metabolism of APL patients. Interestingly, we found that PPAR agonists can reduce arsenic/ATRA-induced dyslipidemia in APL patients and decrease TRIB3 expression in APL cells. This effect may derive from the fact that PPAR agonists inhibit TGFβ1/Smad3 signaling 52 and that *TRIB3* is a target gene of TGFβ1/Smad3 53. Thus, the beneficial effects of PPAR agonists in APL may be derived from not only the direct lowering of blood lipids but also a potential inhibition of TRIB3 expression; this concept needs further clarification.

In summary, our study not only reveals a critical role of the PML-RARα/PPARγ/TRIB3 axis in dyslipidemia for patients with APL but also confers a rationale for the combination of ATRA/arsenic with the PPAR activator for the treatment of patients with APL.

## Supplementary Material

Supplementary figures and tables.

## Abbreviations

APL: acute promyelocytic leukemia
ATRA: all-trans retinoic acid
AML: acute myelogenous leukemia
BMI: body mass index
DEGs: differentially expressed genes
ER: endoplasmic reticulum
FN: fenofibrate
GSEA: gene set enrichment analysis
HDL: high-density lipoprotein
KEGG: kyoto encyclopedia of genes and genomes
LDL: low-density lipoprotein
PPARγ: peroxisome proliferator-activated receptor-γ
PPRE: PPAR-response element
PZD: pioglitazone
RARs: Retinoic acid receptors
TRIB3: tribbles homologue 3
TC: total cholesterol
TG: triglyceride
