# Supplementary material for: PML-RARα interaction with TRIB3 impedes PPARγ/RXR function and triggers dyslipidemia in acute promyelocytic leukemia
Source: Theranostics. 2020 Aug 15;10(22):10326–40. doi: 10.7150/thno.45924 (PMC7481410; doi:10.7150/thno.45924)
Supplement: Supplementary file 1 — Supplementary figures and tables. [file thnov10p10326s1.pdf]

Supplemental Methods

Cell lines and culture

NB4, U937 and C1498 AML cells (mouse non-APL acute myeloid leukemia cells) were purchased from Shanghai Bioleaf Biotech Co., Ltd, where they were recently authenticated by STR profiling and characterized by mycoplasma and cell vitality detection. These cells were cultured and maintained in RPMI 1640 medium supplemented with 10% fetal bovine serum (FBS, Invitrogen, CA, USA) under 5% carbon dioxide.

Leukemia cell transplantation

Mouse APL-like spleen cells from *hMRP8-Pml-Rarα* transgenic mice or normal spleen cells were intravenously injected into nonirradiated FVB mice (2×10^6 cells per mouse) aged between 6 and 10 weeks. To compare the effects of the mouse APL cells and non-APL leukemia cells on the lipid metabolism of the recipient mice, APL cells from *hMRP8-Pml-Rarα* mice or C1498 (non-APL) leukemia cells were intravenously injected into NSG mice (2×10^6 cells per mouse) aged between 4 and 6 weeks. One week later, transplanted mice were sacrificed and analyzed for lipid levels once a week for 4 weeks.

ATRA, arsenic and FN treatment in vivo

APL cells from *hMRP8-Pml-Rarα* transgenic mice were intravenously injected into nonirradiated FVB mice (2×10^6 cells per mouse) aged between 6 and 10 weeks. Mice implanted with leukemic cells were randomly assigned to either type of treatment. One day later, when transplanted cells had established, 400 mg/kg FN (100 μL, p.o., once a day), 2

mg/kg ATRA (100 μL, p.o., once a day) and 2 mg/kg As_2O_3 (100 μL, i.p., once a day), or 400 mg/kg FN (100 μL, p.o., once a day), 2 mg/kg ATRA (100 μL, p.o., once a day) and 2 mg/kg As_2O_3 (100 μL, i.p., once a day) were administered for 3 consecutive weeks. To evaluate the survival rate, these mice were monitored for 28 days.

Chromatin immunoprecipitation (ChIP)-qPCR assay

ChIP assays were performed according to the manufacturer's protocol using a SimpleChIP® Plus Sonication Chromatin IP Kit (Cell Signaling Technology, Danvers, MA, USA, #56383). Briefly, NB4 cells transfected with Myc-tagged PML-RARa plasmids were fixated with 1% formaldehyde for 8 min, incubated with glycine (50 mM final) for 10 min and washed three times with PBS. After cell lysis and chromatin extraction, chromatin was sonicated to 100-500 bp using a BioRuptor sonicator (Diagenode), followed by centrifugation at 16,000 g for 10 min at 4 °C. The lysates were incubated overnight at 4 °C with ChIP-grade antibodies specific for Myc (Abcam, #ab9132), which were coupled to magnetic beads. Precipitated material was eluted (input chromatin was used as a control), the crosslink was reverted, and DNA was purified by chloroform/phenol extraction and resuspended in DNA elution buffer. qPCR analysis was performed using specific primers corresponding to the TRIB3 and PPARG promoters. The primers used for the ChIP-qPCR assays are shown below: TRIB3-1-F: 5'-CCCCACAACTTATATCTAGTGCAGG-3', TRIB3-1-R: 5'-CAGCTGGCATTTAG GGAGCATGTCT-3'; TRIB3-2-F: 5'-TCCCTAAATGCCAGCTGTTATTATG-3', TRIB3-2-R: 5'-CATGGACAAGCGCTTGTCTCTCACT-3'; TRIB3-3-F: 5'-TGAGAGACAAGCGC

TTGTCCATGCC-3', TRIB3-3-R: 5'-AGAATCTGTGTGGTCATTTCTG CAT-3'; TRIB3-4-F: 5'-AATGACCACACAGATTCTACCAATG-3', TRIB3-4-R: 5'-TGTC TGTGAGGTCTT CAGAGCTGAT-3'; TRIB3-5-F: 5'-AGCTCTGAAGACCTCACAGACA CAT-3', TRIB3-5-R: 5'-GCTGAGATTACAGGCGTGAGCCACA-3'; TRIB3-6-F: 5'-TGTAATCTCAG CGCTTTGGGAGGCC-3', TRIB3-6-R: 5'-GCTGGAGTGCAATGGCGTGATCTTG-3'; TRIB3-7-F: 5'-TTGCACTCCAGCCTGGGTGACAGAA-3'; TRIB3-7-R: 5'-GGCCATGC CATGTCCAAGGTCACAG-3'; TRIB3-8-F: 5'-ATTCCCTGTGAC CTTGGACATGGCA-3', TRIB3-8-R: 5'-AGAGCTTGGTTTTGAGCCATGTGCT-3'; TRIB3-9-F: 5'-ACATGGCTCA AAACCAAGCTCTGGG-3', TRIB3-9-R: 5'-TGCCACACCTGGTCCATGGACCCTG-3'; TRIB3-10-F: 5'-AGGGTCCATGGACCAGGTGTGGCAG-3', TRIB3-10-R: 5'-AGGATGG AAGCAAAGCTGCAGCCCT-3'; PPARG-1-F: 5'-ACATTGCTGGTGGGATTGTAAA ATG-3', PPARG-1-R: 5'-TCCATTATACACATATACCACATTT-3'; PPARG-2-F: 5'-ATGTGGTATATGTGTATAATGGAAT-3', PPARG-2-R: 5'-ACCCCCTCTCCATTA ACAGTCATTC-3'; PPARG-3-F: 5'-ATGACTGTTAATGGAGAGGGGGTTC-3', PPARG-3-R: 5'-ATGACTGTTAATGGAGAGGGGGTTC-3'; PPARG-4-F: 5'-AGGGTCAAGCG ATTCTACTGCCTCA-3', PPARG-4-R: 5'-TACAATTCAGGCCGGGTATGGCAGC-3'; PPARG-5-F: 5'-ACCCGGCCTGAATTGTACATTT TAC-3', PPARG-5-R: 5'-TAATTTTAA TTGTTTAGTAGAGACT-3'; PPARG-6-F: 5'-AACATGTCAAGACACAGTCTCTACT-3', PPAR G-6-R: 5'-TCTTTTCTTTCTTTCTTCCATGAGA-3'; PPARG-7-F: 5'-AAAGAAAGAAAAGAAAGGAAGAAAG-3', PPARG-7-R: 5'-GTCCTTCCTCCAC AGCCCCTAAGAT-3'; PPARG-8-F: 5'-CTGTGGAGGAAGGACATGATTATGT-3',

PPARG-8-R: 5'-TTCTGGGCCTGATCCTCTTTGGGGA-3'; PPARG-9-F: 5'-AGGATCAGG CCCAGAACAGTATGCT-3', PPARG-9-R: 5'-AGGCA CGAGAAACAGTTTCTCATGT-3'; PPARG-10-F: 5'-AAACTGTTTCTCGTGCCTCACGTCC-3', PPARG-10-R: 5'-ACATGG TTATTCACAAGTCA CTGAC-3'.

In vivo study of PPARγ function

To generate cells stably expressing PPARγ-shRNA, PPARγ-shRNA viral particles were purchased from Genecopoeia, and the target sequence of the shRNA was 5'-CAACAGGCCTCATGAAGAA-3'. After 24 h of infection, PPARγ-shRNA stable C1498 cells were selected in medium containing 1 μg/mL puromycin for 14 days. After 2-3 passages in the presence of puromycin, the cultured cells were used for experiments without cloning. Control-shRNA or PPARγ-shRNA C1498 cells (2 x 10^6) were injected intravenously into C57BL/6 mice aged between 6 and 8 weeks. One week later, transplanted mice were sacrificed and analyzed for lipid levels once a week for 4 weeks.

Measurement of liver lipids

The fresh mouse liver was isolated and ground uniformly, and 100 mg of liver tissue was accurately weighed into the EP tube. Then, 1 mL of a chloroform/methanol solution (chloroform: methanol = 2:1, V/V) was added, followed by vigorous vortexing of the mixture for 1 min and letting it stand for 2 h on ice. Then, the mixture was centrifuged at 1650 g for 10 minutes at 4 °C. The mixture was separated into 3 layers: the upper methanol layer, the middle

protein disc, and the bottom chloroform and lipids. The bottom phase was transferred to a new EP tube and stored at -80 °C before use. The total TG and TC levels were measured by using commercial kits according to the manufacturers' protocols.

Isolation of primary mouse hepatocytes

Primary hepatocytes were isolated from normal mouse livers as described in JoVE [1]. Briefly, mouse livers were perfused in situ with EGTA solution followed by pronase (Sigma, P5147) solution and collagenase D (Roche, 11088882001) solution at 37 °C for primary hepatic cell isolation. After perfusion, the liver was transferred into a sterile Petri dish containing protease and collagenase D solution. After being gently minced, the liver and cell suspension were filtered through a 70-μm cell strainer and centrifuged at 300 g for 3 min at 4 °C to isolate hepatocytes. Primary hepatocytes were cultured in DMEM (Gibco, 10564029) supplemented with 10% FBS, insulin (Sigma, I9278), dexamethasone (Sigma, D4902) and penicillin-streptomycin (Gibco, 15070063) on collagen-coated dishes (Corning, 354236).

Coculture of APL cells with hepatocytes

For coculture, primary mouse hepatocytes were preseeded in 24-well plates and cultured with DMEM supplemented with 10% FBS, insulin, dexamethasone and penicillin-streptomycin on collagen-coated dishes overnight. Primary isolated APL or AML cells (5×10^4 cells/well in 10% FBS in RIPM 1640) were added to the transwell (STEMCELL, #38024) upper chamber, and hepatocytes and leukemia/normal spleen cells were added at a ratio of 10:1. The

leukemia/normal cells and hepatocytes were cocultured together at 37 °C for 48 h and then separated for further analysis.

ELISA and TC/TG measurement

Human resistin and leptin levels in plasma or culture supernatant were determined by enzyme-linked immunosorbent assay (ELISA) according to the manufacturer's instructions (Beijing 4A Biotech Co., Ltd, China). Mouse PCSK9 levels in plasma or culture supernatant were determined by ELISA according to the manufacturer's instructions (Sino Biological, Beijing, China). Cholesterol and triglycerides (TGs) in the cell culture were assessed by an enzymatic assay according to the supplier's protocols (Applygen Technologies Inc., Beijing, China).

Stable cell lines

To generate cells stably expressing TRIB3-shRNA1/2 (T3 sh) or control-shRNA (CTRL), plasmids were transfected into NB4 cells with Lipofectamine LTX with Plustransfection reagent (Invitrogen, CA, USA) according to the manufacturer's instructions. After 24 h of transfection, stable transfectants were selected in medium containing 1 μg/mL puromycin (Gibco, CA, USA) for 14 days. After 2-3 passages in the presence of puromycin, the cultured cells were used for experiments without cloning. To generate cells stably expressing *TRIB3*^{Cas9}, *TRIB3*^{Cas9} viral particles were purchased from TransOMIC Technologies Inc., and the gRNA target sequence was 5'-GTGCTGGTGACAGTGCGCCA-3'. Cas9 then cut at this sequence, and nonhomologous end-joining (NHEJ) took place. TRIB3-C (CRISPR Clone ID) was used

to construct stable *TRIB3*^{Cas9} NB4 cells. After 24 h of infection, stable cells were selected in medium containing 1 μg/mL puromycin for 14 days. After 2-3 passages in the presence of puromycin, the cultured cells were used for experiments without cloning.

Flow cytometry

Fluorescently labeled antibodies against the following surface proteins were used for human cell staining: CD11b (human), annexin-V FITC, and proprium iodide (PI). Data were acquired using a FACS^{Canto} II flow cytometer (BD, CA, USA). FCS Express software was used for data analysis.

Immunoprecipitation, immunoblotting, and immunostaining

Co-IP experiments were performed as described previously[23]. Briefly, cells were collected and lysed for 30 min on ice. Soluble lysates were incubated with the indicated antibodies at 4 °C overnight, followed by incubation with Protein A/G Plus-Agarose (Santa Cruz Biotechnology, TX, USA) at 4 °C for 2 h. Immunocomplexes were separated from the beads and then boiled for 10 min. The precipitated proteins were subjected to SDS-PAGE and blotted with specific antibodies. For immunoblotting assays, proteins were extracted from cells using RIPA buffer (Cell Signaling Technology, MA, USA). A BCA Protein Assay Kit was used to determine protein concentrations. Protein extracts were separated by SDS-PAGE, transferred onto PVDF membranes, and subjected to immunoblot analysis. Western blot images were captured by a Tanon 5200 chemiluminescent imaging system (Tanon, Shanghai, Beijing).

Real-time PCR and RNA interference

Total RNA was extracted using TRIzol (Invitrogen, CA, USA) according to the manufacturer's instructions. Reverse transcription of the total cellular RNA was carried out using oligo (dT) primers and M-MLV reverse transcriptase (Transgen Biotech, Beijing, China). PCR was performed using a Mycycler thermal cycler and analyzed using agarose gels. The sequences of the PCR primers were as follows: *PPARG* forward, 5′-TCTCTCCGTAATGGAAGACC-3′; *PPARG* reverse, 5′-CCCCTACAGAGTATTACG-3′; *RETN* forward, 5′-AGCCATCAATGAGAGGATCCAG-3′; *RETN* reverse, 5′-TCCAGGCCAATGCTGCTT-3′; *GPHN* forward, 5′-TCGCCTCTCTACAGCTTCCT-3′; *GPHN* reverse, 5′-CTGCACCTGGACTGGACATT-3′; *ME1* forward, 5′-CGGAACCCTCACCTCAACAA-3′; *ME1* reverse, 5′-GTTGAAGGAAGGTGGC AACA-3′; *LEP* forward, 5′-ATGCATTGG GGAACCCTGTGCGG-3′; *ME1* reverse, 5′-TGAGGTCCAGCTGCCACAGCATG-3′; *LTC4S* forward, 5′-ACGAGGTAGCTCTACTGGCTG-3′; *LTC4S* reverse, 5′-ACCTGCAGGGAGAAGTAGGC-3′; *DHCR7* forward, 5′-GCCATGGTCAAGGGCTACTT-3′; *DHCR7* reverse, 5′-ACTTCCCGA TCCGAGGGTTA-3′; *NKIRAS1* forward, 5′-GCTGCAAGGTTGTGGTTTGT-3′; *NKIRAS1* reverse, 5′-CACGCCTTCCTGTAGACCTC-3′; *MOSC2* forward, 5′-GCAAGCAGCCTTCCTCAAAC-3′; *MOSC2* reverse, 5′-GCCTCATTGCCACAGTCTCT-3′; *ABCA1* forward, 5′-ATGGCTTGTTGGCCTCAGC-3′; *ABCA1* reverse, 5′-GCAGCAGCTGACATGTTTGT-3′; *GAPDH* forward, 5′-GTGGACATCCGCAAAGACC-3′; and *GAPDH* reverse, 5′-CCTAGAAGCATTTGCGGTG-

3′. *TRIB3* siRNAs were produced by RiboBio (Guangzhou, China) and transfected using Lipofectamine RNA interference MAX Transfection Reagent (Life Technologies, CA, USA) according to the manufacturer's instructions.

Gene set enrichment analysis (GSEA)

We ranked the 11,378 genes by their association with the APL (n = 50) and non-APL (n = 50) groups (GSE13204) using the signal-to-noise measure in the GSEA program according to log2-fold changes. Lipid metabolic gene sets were collected from the database (http://software.broadinstitute.org/gsea/msigdb/index.jsp).

Antibody table

ANTIBODY	SOURCE	IDENTIFIER
Anti-TRIB3 antibody	Abcam	ab75846
Anti-TRIB3 antibody	Abcam	ab137526
PPARγ antibody	CST	C26H12
Anti-leptin antibody	Abcam	Ab3583
Anti-resistin antibody	Abcam	Ab124681
Gapdh	Zsjqbio	Ta-08
Anti-PML protein antibody	Abcam	Ab96051
PML protein antibody	Novus	NB100-59787
RXRα/β/γ antibody	Santa Cruz	sc-46659
Anti-Myc-tag mAb	MBL BIOTECH	M047-3
Anti-Flag-tag mAb	MBL BIOTECH	PM020
Ubiquitin antibody	CST	3933S
Anti-retinoic acid receptor alpha antibody	Abcam	ab28767
β-Actin (D6A8) rabbit mAb	CST	8457S
Anti-Myc magnetic beads	Biotool	B26302
Anti-Flag magnetic beads	Biotool	B26102
ALEXA FLUOR(R) 488 RABBIT	Invitrogen	A21210
Mounting medium with DAPI	Zsbio	ZLI-9557

Donkey anti-rabbit IgG (H+L) highly cross-adsorbed secondary antibody, Alexa Fluor 647	Invitrogen	A31573
Donkey anti-mouse IgG (H+L) highly cross-adsorbed secondary antibody, Alexa Fluor 555	Invitrogen	A31570
Alexa Fluor® 647 anti-mouse/human CD11b	Biolegend	101220

Supplementary Figures and Legends

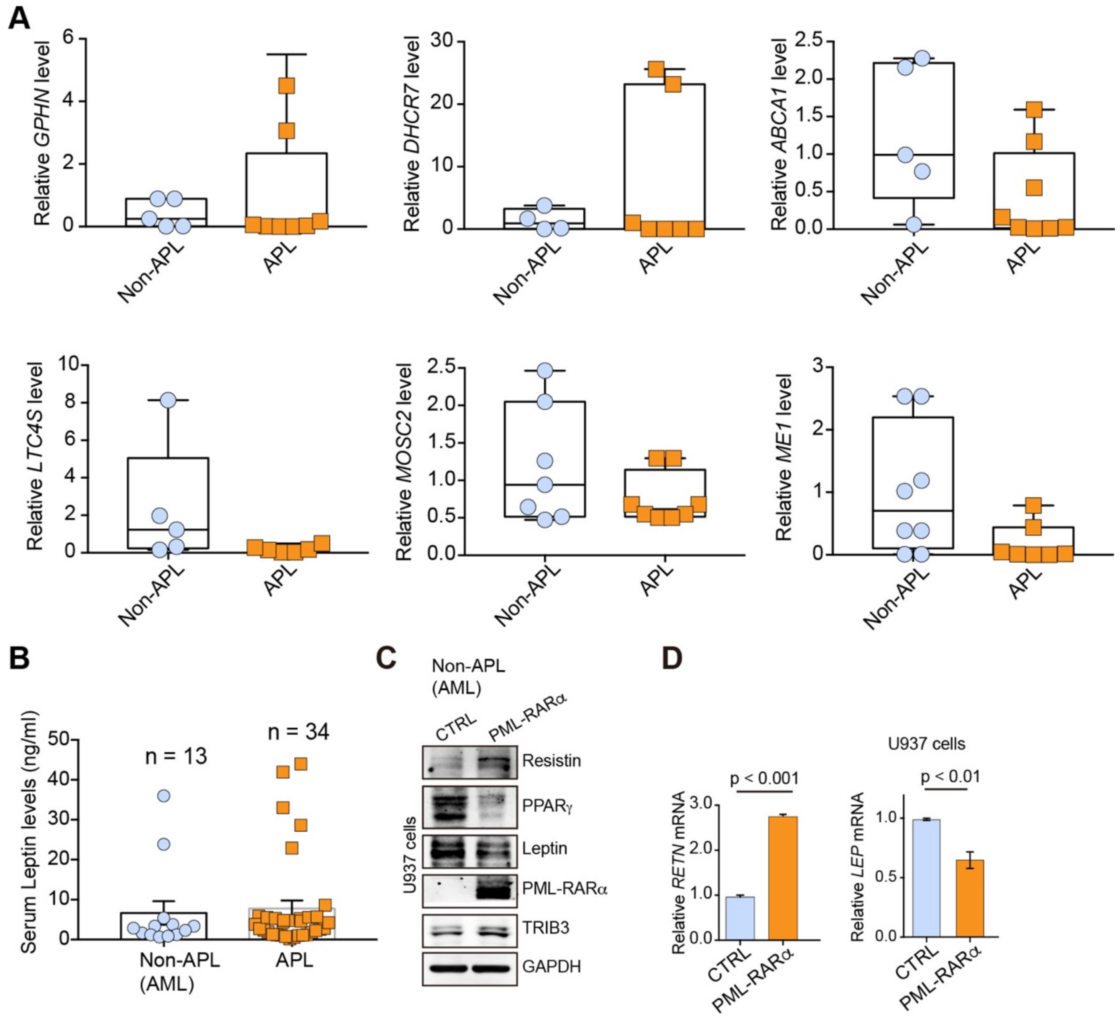

Supplementary Figure S1. (A) qRT-PCR was performed to analyze the mRNA levels of *GPHN*, *DHCR7*, *ABCA1*, *LTC4S*, *MOSC2*, and *ME1* in primary APL cells and non-APL AML cells. **(B)** Serum leptin levels in newly diagnosed APL patients (n = 34) and non-APL AML patients (n = 13). **(C)** The effects of PML-RARα overexpression on the protein levels of PPARγ, resistin, leptin, and TRIB3 in U937 cells. **(D)** The effects of PML-RARα overexpression on the mRNA levels of *RETN* and *LEP* in U937 cells. The indicated mRNA levels in U937 cells with or without PML-RARα overexpression were detected by qRT-PCR.

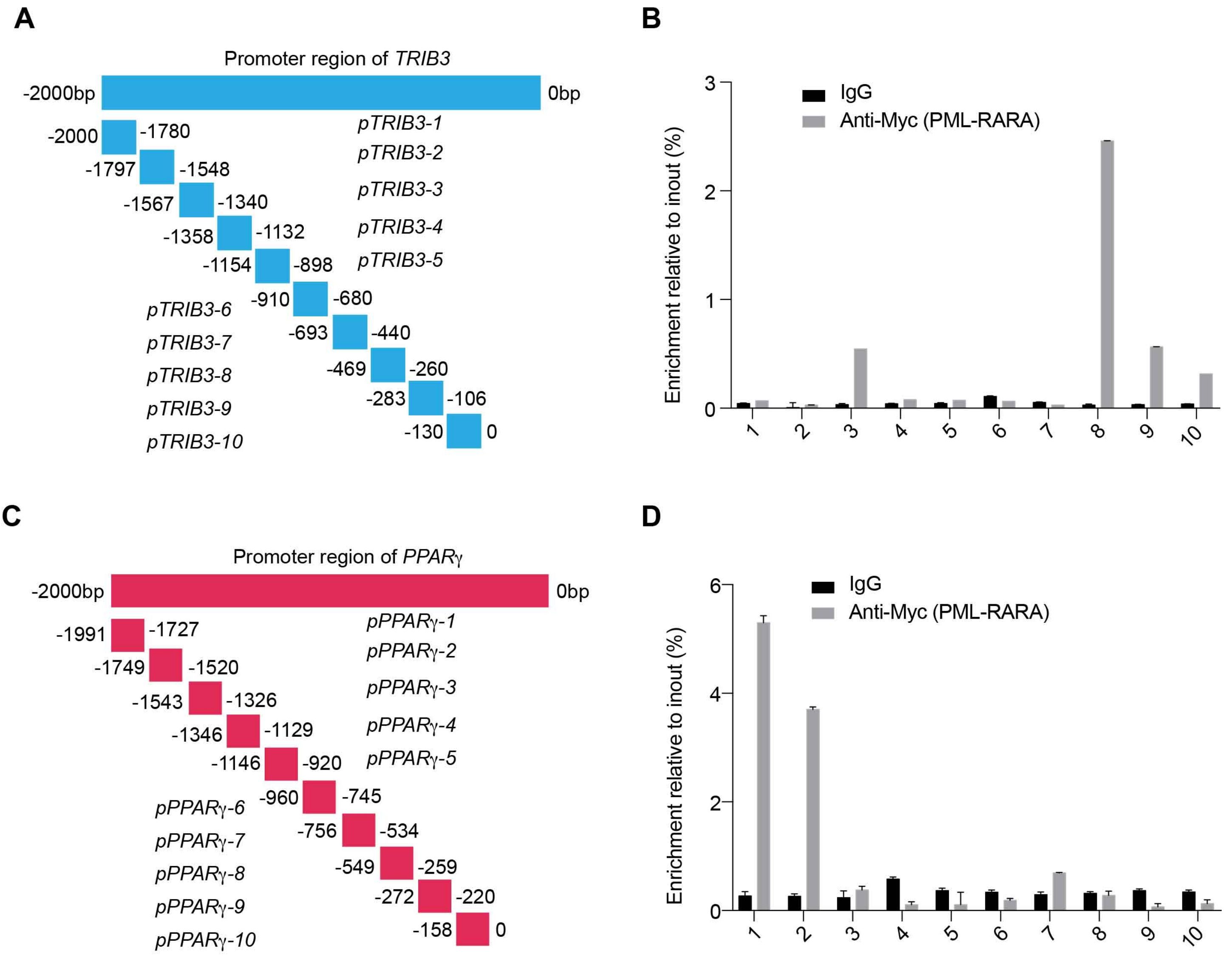

Supplementary Figure S2. TRIB3 and PPARG genes are direct targets of PML-RARa. **(A)** The TRIB3 promoter was analyzed. Fragments located upstream of ATG were used as the promoter region. **(B)** ChIP-qPCR assays were performed using different specific primers corresponding to the TRIB3 promoter region. **(C)** The PPARG promoter was analyzed. Fragments located upstream of ATG were used as the promoter region. **(D)** ChIP-qPCR assays were performed using different specific primers corresponding to the PPARG promoter region.

References

1. Au - Cui A, Au - Hu Z, Au - Han Y, Au - Yang Y, Au - Li Y. Optimized Analysis of In Vivo and In Vitro Hepatic Steatosis. *JoVE.* 2017(121):e55178.

Table S1. Clinical information of patients samples used in Figure 1

Patient No	APL = 1; non-APL AML = 2	Gender	Age	Height (cm)	Body weight (kg)	BMI>25= 1; <25=0	BMI
1	1	Male	45	168	77	1	27.281746
2	1	Female	51	162	53	0	20.1950922
3	1	Female	37	163	50	0	18.8189243
4	1	Male	19	176	70	0	22.5981405
5	1	Female	30	163	47	0	17.6897889
6	1	Female	29	156	65	1	26.7094017
7	1	Female	25	160	54	0	21.09375
8	1	Male	38	172	69	0	23.3234181
9	1	Female	61	162	55	0	20.9571712
10	1	Female	52	161	65	1	25.076193
11	1	Female	34	160	60	0	23.4375
12	1	Female	15	160	50	0	19.53125
13	1	Male	52	179	90	1	28.089011
14	1	Female	61	157	60	0	24.3417583
15	1	Female	44	162	60	0	22.8623685
16	1	Male	34	180	85	1	26.2345679
17	1	Male	27	178	107	1	33.7709885
18	1	Male	66	173	70	0	23.3886866
19	1	Female	26	160	55	0	21.484375
20	1	Female	34	160	62	0	24.21875
21	1	Female	13	156	45	0	18.4911243
22	1	Male	29	170	65	0	22.4913495
23	1	Female	24	153	72	1	30.757401
24	1	Female	21	165	60	0	22.0385675
25	1	Female	29	170	73	1	25.2595156
26	1	Male	33	168	72	1	25.5102041
27	1	Male	41	175	75.5	0	24.6530612
28	1	Male	39	168	69	0	24.4472789
29	1	Male	57	170	74	1	25.6055363
30	1	Male	34	170	81	1	28.0276817
31	1	Female	44	162	54	0	20.5761317
32	1	Female	30	160	54	0	21.09375
33	1	Female	61	154	60	1	25.2993759
34	1	Male	49	165	71	1	26.0789715
35	1	Female	62	156	76	1	31.2294543
36	1	Female	63	172	70	0	23.6614386
37	1	Male	43	180	78	0	24.0740741

38	1	Male	47	170	77	1	26.6435986
39	1	Female	45	162	75	1	28.5779607
40	1	Male	45	170	80	1	27.6816609
41	1	Female	28	160	75	1	29.296875
42	1	Female	29	165	57.5	0	21.1202938
43	1	Male	44	162	67.5	1	25.7201646
44	1	Male	54	167	65	0	23.3066801
45	1	Female	16	152	65	1	28.1336565
46	1	Male	41	168	74	1	26.2188209
47	1	Male	33	173	59	0	19.7133215
48	1	Female	19	163	68	1	25.5937371
49	1	Male	40	165	62	0	22.7731864
50	1	Male	30	174	94	1	31.0476945
51	1	Male	47	160	61	0	23.828125
52	1	Male	27	177	95	1	30.3233426
53	1	Male	46	160	72	1	28.125
54	1	Female	17	165	62	0	22.7731864
55	1	Male	50	170	78	1	26.9896194
56	1	Male	32	174	78	1	25.7629806
57	1	Male	59	174	81	1	26.7538644
58	1	Male	26	179	95	1	29.6495116
59	1	Male	17	171	80	1	27.3588455
60	1	Male	28	175	80	1	26.122449
61	2	Male	50	168	77	1	27.281746
62	2	Male	37	178	77	0	24.3024871
63	2	Female	29	166	49	0	17.7819713
64	2	Male	39	178	79	0	24.9337205
65	2	Female	48	165	58	0	21.3039486
66	2	Male	48	176	91	1	29.3775826
67	2	Male	55	168	70	0	24.8015873
68	2	Female	57	160	48	0	18.75
69	2	Female	53	165	49	0	17.9981635
70	2	Male	43	170	70	0	24.2214533
71	2	Male	59	169	75.5	1	26.4346486
72	2	Male	26	181	65	0	19.8406642
73	2	Male	43	172	81	1	27.3796647
74	2	Male	17	183	100	1	29.8605512
75	2	Male	24	165	61	0	22.405877
76	2	Male	45	174	73.5	0	24.2766548
77	2	Male	24	180	95	1	29.3209877
78	2	Male	42	172	90	1	30.4218496
79	2	Female	28	150	53	0	23.5555556

80	2	Male	54	170	77	1	26.6435986
81	2	Male	38	176	95	1	30.668905
82	2	Female	17	150	61	1	27.1111111
83	2	Female	49	158	55	0	22.0317257
84	2	Female	26	162	55	0	20.9571712
85	2	Male	54	183	97	1	28.9647347
86	2	Female	39	160	66	1	25.78125
87	2	Male	41	165	65	0	23.8751148
88	2	Male	39	174	72	0	23.7812128
89	2	Male	57	155	54	0	22.4765869
90	2	Male	51	165	65	0	23.8751148
91	2	Male	45	170	85	1	29.4117647
92	2	Female	45	166.5	63	0	22.7254281
93	2	Male	21	167	56	0	20.0796013
94	2	Female	43	159	59	0	23.3376844
95	2	Male	42	159	48.5	0	19.1843677
96	2	Female	25	165	70	1	25.7116621
97	2	Female	22	160	54	0	21.09375
98	2	Male	52	176	95	1	30.668905
99	2	Male	49	165	58.5	0	21.4876033
100	2	Female	33	165	66	0	24.2424242
101	2	Male	24	172	57	0	19.2671714
102	2	Female	59	168	55	0	19.4869615
103	2	Male	25	188	95	1	26.8786781
104	2	Male	29	175	80	1	26.122449
105	2	Female	23	165	60.5	0	22.2222222
106	2	Female	55	168	55	0	19.4869615
107	2	Male	31	181	81	0	24.72452
108	2	Female	26	164	60	0	22.3081499
109	2	Male	37	168	62.5	0	22.1442744
110	2	Female	43	159	70	1	27.6887781
111	2	Male	43	165	55	0	20.2020202
112	2	Female	31	167	50.5	0	18.1074976
113	2	Female	61	158	52	0	20.8299952
114	2	Female	28	170	56	0	19.3771626
115	2	Male	45	170	75	1	25.9515571
116	2	Male	44	172	62	0	20.9572742
117	2	Female	38	172	64	0	21.6333153
118	2	Female	25	170	59	0	20.4152249
119	2	Female	62	159	52	0	20.5688066
120	2	Female	19	165	62	0	22.7731864

Table S2. APL patient treatment history and sample details, related to Figure 2, 3, 5, 6, and 7.

Name	AML types	Sample type	Gender	Age	Treatment history
1	APL	PB+BM	M	27	ATRA + Arsenic
2	APL	PB+BM	F	24	Ara-C + ATRA
3	APL	PB+BM	M	62	ATRA + Arsenic
4	APL	PB+BM	M	41	ATRA + Arsenic
5	APL	PB+BM	M	20	ATRA + Arsenic
6	APL	PB+BM	F	49	ATRA + Arsenic
7	APL	PB+BM	M	40	ATRA + Arsenic
8	APL	PB+BM	M	34	ATRA + Arsenic
9	APL	PB+BM	M	34	ATRA + Arsenic
10	APL	PB+BM	F	49	ATRA + Arsenic
11	APL	PB+BM	M	34	ATRA + Arsenic
12	APL	PB+BM	M	44	ATRA + Arsenic
13	APL	PB+BM	M	33	ATRA + Arsenic
14	APL	PB+BM	M	38	ATRA + Arsenic
15	APL	PB+BM	F	41	IA, CAG
16	APL	PB+BM	M	31	CAG
17	APL	PB+BM	M	20	CAG
18	APL	PB+BM	M	38	ATRA
19	APL	PB+BM	F	33	IA
20	APL	PB+BM	M	18	IA
21	APL	PB+BM	M	38	ATRA
22	APL	PB+BM	F	61	HAA
23	APL	PB+BM	M	27	ATRA
24	APL	BM	F	54	HAA
25	APL	PB	M	53	ATRA
26	APL	PB	F	61	HAA
27	APL	BM+PB	M	27	ATRA
28	APL	BM	F	48	ATRA
29	APL	BM	F	16	ATRA
30	APL	BM	F	50	ATRA
31	APL	BM	F	35	ATRA
32	APL	BM	M	58	ATRA
33	APL	BM	F	17	ATRA
34	APL	BM	M	23	Dasatinib
35	APL	BM	F	30	ATRA
36	APL	BM	F	48	ATRA
37	APL	BM	M	58	ATRA
38	APL	BM	M	55	ATRA + Sorafenib
39	APL	BM	F	17	ATRA
40	APL	BM	F	35	ATRA
41	APL	BM	F	16	ATRA
42	AML-M2 ETO+ K	PB	M	48	HAA
43	AML-ETO	BM	M	20	IA, HAA
44	AML	PB	F	19	
45	AML-ETO	BM	M	21	
46	AML-ETO	BM	M	48	HAA
47	B-ALL	PB	M	46	Dasatinib
48	AML (FLT3-ITD)	PB	M	50	Sorafenib
49	AML (FLT3-ITD)	PB	F	68	Sorafenib

50	AML	PB	F	55	HAA
51	non-APL	PB	M	18	CAG
52	non-APL	PB	F	54	HAA
53	T-ALL	PB	M	29	CAG
54	non-APL (FLT3-I[illegible]	BM	F	15	Dasatinib

APL, Acute Promyelocytic Leukemia; AML, Acute Myelogenous Leukemia; PB, Peripheral Blood; BM, Bone Marrow; F, Female; M, Male; ATRA, All-trans retinoic acid; HAA, Homoharringtonine combined aclarubicin and cytarabine; CAG, Cytarabine, Aclarubicin and G-CSF; IA, Idarubicin.
